# Performance Analysis and Optimization of a Venturi-Type Hydrogen–Natural Gas Mixer

**DOI:** 10.3390/e28080888

**Published:** 2026-08-06

**Authors:** Pinru Chen, Fengyun Li, Jun Zheng, Weiqing Xu

**Affiliations:** 1 School of Automation Science and Electrical Engineering, Beihang University, Beijing 100191, China; pinru.chen@buaa.edu.cn; 2 Wuhan Second Ship Research Institute, Wuhan 430064, China; lifengyun@buaa.edu.cn

**Keywords:** static mixer, hydrogen blending, Venturi-type mixer, computational fluid dynamics, mixing uniformity

## Abstract

Blending hydrogen into existing natural-gas pipeline networks provides a practicable route toward future low-carbon applications. A Venturi-type mixer is a classical high-efficiency static gas-mixing device, and clarifying the effects of its structural parameters is important for efficient transport and downstream combustion stability. In this study, numerical simulations were performed in ANSYS Fluent 2024 R1. The contraction angle, throat length, and diffuser angle were selected as representative structural variables. First, the independent effects of these variables on the mixing process were examined through single-factor simulations. Then, three key levels of the three structural parameters were selected to establish a Box–Behnken experimental matrix for response-surface modeling. Based on the numerical results, entropy weighting and a genetic algorithm were used for multi-objective optimization, and the final solution was verified using the TOPSIS method. The results show that the optimized Venturi-type mixing device with optimized parameters of a contraction angle of 20.7°, a throat length of 60 mm, and a diffuser angle of 5° can reduce flow energy loss while maintaining high mixing uniformity. The diffuser angle is the dominant geometric parameter affecting both energy loss and mixing behavior. Compared with the reference central-point structure design, the overall TOPSIS score of the optimized structure increased from 0.41 to 0.82; the pressure loss decreased from 258.94 Pa to 206 Pa, corresponding to a reduction of approximately 20%; and the final-section mixing uniformity decreased only slightly, from 97.85% to 97.43%.

## 1. Introduction

To address global climate change, hydrogen-enriched natural gas has been widely considered a transitional fuel strategy with both engineering feasibility and emission-reduction potential. Compared with constructing pure-hydrogen combustion systems, hydrogen blending can partly utilize mature gas-distribution infrastructure. The key concept is to add a certain fraction of hydrogen to natural gas, thereby reducing the carbon content per unit heating value and improving combustion reactivity, which helps reduce CO_2_, CO, and other carbon-containing emissions. However, hydrogen has low density, a high diffusion coefficient, low ignition energy, and a high laminar burning velocity. After hydrogen blending, fuel properties, mixing behavior, and flame-propagation characteristics can be altered [1] depending on the blending ratio, which can affected flame-propagation and stability-related parameters; this introduces new technical and safety challenges. For example, metallic pipelines are susceptible to hydrogen embrittlement in hydrogen-rich environments [2]. Therefore, hydrogen-enriched natural gas is not a simple fuel-substitution issue, but a multi-physics problem involving fuel transport, mixture uniformity, combustion stability, and pollutant formation. Previous studies have shown that uniform transport can reduce the likelihood of hydrogen embrittlement in pipelines [3]. Sorgulu et al. found that natural gas blended with 10–30% hydrogen by volume can be used in practical combustion tests and changes gas consumption, heating time, and heating-value utilization [4]. Excessive hydrogen blending can increase fuel-quality deterioration and explosion risk; however, such risks can be mitigated by monitoring equipment [5]. Zhao et al. suggested that limiting the hydrogen fraction to 25% improves transport safety and reliability [6]. Hence, improving mixture uniformity during pipeline transport is necessary.

From the perspective of combustion emissions, adding hydrogen to natural gas enhances the reactivity of the blended fuel. Studies have shown that as the hydrogen fraction increases, the laminar burning velocity of methane- or natural gas-based mixtures increases significantly, which can broaden the lean-burn stability range and reduce unburned hydrocarbons and CO emissions [7,8,9]. Nevertheless, the high burning velocity and preferential diffusion of hydrogen can also increase the risks of flashback, local overheating, and combustion oscillations. In premixed combustion systems, upstream mixing non-uniformity may generate local hydrogen-rich or near-stoichiometric regions at the outlet, which can induce local high-temperature zones and increase NOx formation. Sun et al. reported that hydrogen addition under gas turbine-relevant operating conditions can inhibit CO formation but may increase NO emissions; therefore, pollutant control requires a lower equivalence ratio and improved mixing uniformity [10].

In pipeline transport and mixing engineering, the mixer is the key component used to ensure uniformity. It must realize rapid and homogeneous mixing of natural gas and hydrogen while controlling pressure drop and flow energy loss. Insufficient mixing may cause non-uniform outlet composition and unstable flame structures in downstream devices, whereas excessive mixing enhancement may lead to high total-pressure loss and reduced energy efficiency. Studies on dry low-emission combustors and lean-premixed gas-turbine combustion have shown that hydrogen addition shortens flame length, shifts the heat-release zone upstream, and modifies thermoacoustic oscillations and combustion stability [11,12]. These findings indicate that the low-emission benefit of hydrogen-enriched natural-gas combustion depends not only on fuel composition but also on flow organization and species distribution in the upstream mixing and transport process.

Current mixing devices can be broadly classified into dynamic and static mixers. Compared with dynamic mixers, static mixers including Venturi-type designs have simple structures, low manufacturing cost, no moving components, controllable pressure drop, and strong mixing-enhancement capability [13]. The fundamental mechanism is that the contraction section accelerates the main flow and forms a low-pressure region at the throat, inducing secondary fluid entrainment. Subsequently, velocity gradients, shear-layer development, and turbulent diffusion in the diffuser promote further species mixing. Studies on industrial premixer and Venturi-type gas mixers have shown that geometric optimization can improve fuel-ratio control and mixture uniformity while helping mitigate NOx-related risks [14]. CFD investigations of Venturi-type mixers for dual-fuel engines have also demonstrated that the throat substantially increases local velocity and turbulence intensity, making the mixture more uniform in the diffuser section [15]. These studies provide a basis for applying Venturi structures to hydrogen-enriched natural-gas premixing.

In response to these challenges, this study proposes a Venturi-type static mixer for blending hydrogen into natural gas. In addition, we conduct a systematic analysis of key structural parameters and fluid parameters to determine the optimal configuration. The main contributions of this work are as follows: First, the classic Venturi-type mixer is introduced into blending application, and its time-averaged flow organization and species-redistribution characteristics are systematically analyzed, providing a new structural design approach for hydrogen injection in pipelines. Second, existing Venturi-mixer studies are mostly concerned with conventional natural gas, biogas, or general gaseous fuels; systematic analyses of the coupled effects among structural parameters, hydrogen blending ratio, mixing uniformity, and pressure loss for hydrogen–natural gas multi-component systems are still limited. Recent studies on static mixers for hydrogen-enriched natural gas have shown that mixer geometry significantly affects concentration uniformity, internal flow organization, and pressure-loss characteristics. These studies commonly evaluate mixer performance using the coefficient of variation in species concentration, pressure drop, and streamline or concentration-field distributions [16,17]. Thus, a quantifiable performance-evaluation method for specific mixing structures is needed.

Based on these considerations, this study investigates a Venturi-type mixing device for low-emission hydrogen-enriched natural-gas blending using computational fluid dynamics. This work establishes a multi-component flow model for natural gas and hydrogen in the Venturi mixer; analyzes the effects of the three primary Venturi parameters—contraction angle, throat length, and diffuser angle—on velocity field, pressure field, turbulence intensity, and species distribution; and evaluates performance using mixing uniformity and total-pressure loss. The trade-off between mixing enhancement and flow loss is revealed. Response-surface methodology, entropy weighting, and a genetic algorithm are then combined to propose an optimized structure that simultaneously achieves high mixing uniformity and low pressure loss. The results provide theoretical guidance for mixer design and operating-parameter selection in hydrogen-enriched natural-gas transporting systems and offer a basis for reducing CO and UHC emissions while controlling NOx formation risks.

## 2. Physical and Mathematical Models

### 2.1. Physical Model

**Figure 1:**
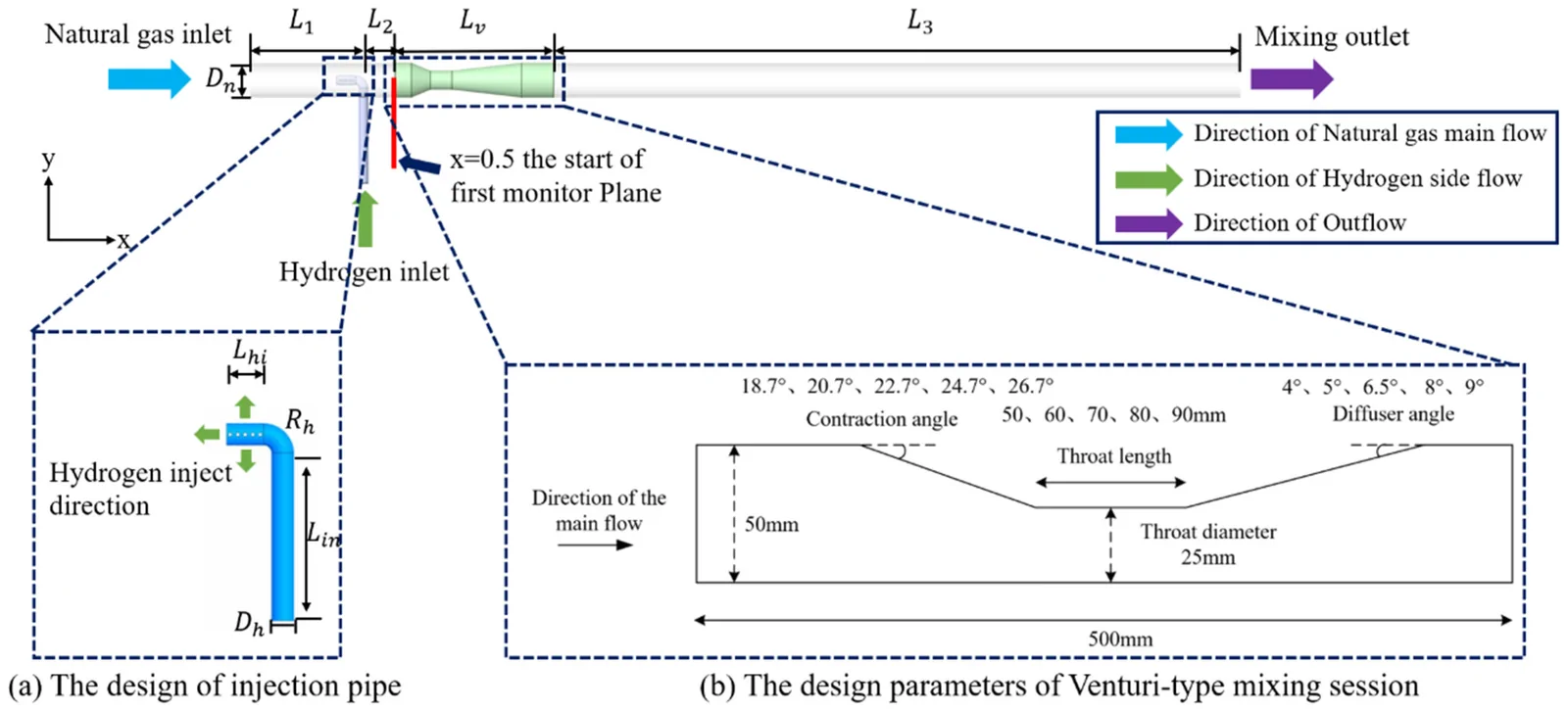
Schematic diagram of different parts of the Venturi-type mixing device: (**a**) The design of injection pipe of Hydrogen; (**b**) The design parameters of Venturi-type mixing session.

Venturi-type static mixers play an important role in fluid mixing because of their simple structure, low manufacturing cost, absence of moving parts, controllable pressure drop, and high mixing efficiency [18]. In this study, the Venturi-type mixer was found to improve the mixing of natural gas and hydrogen effectively while producing relatively low pressure loss compared with other static-mixer configurations [19,20]. As shown in Figure 1, natural gas enters horizontally along the x-direction, hydrogen enters upward along the injection pipe and is mixed with the main stream through a reverse-injection pipe, and the two gases are mixed in the Venturi section before leaving through the outlet. The inlet mixing section, Venturi core, and outlet final-mixing section are arranged in a three-section layout, following related design practices [16,21], so that the remaining model variables remain consistent when the Venturi parameters are changed.

Figure 1a illustrates the hydrogen-injection configuration adopted in the present study. The counter-flow arrangement was not selected arbitrarily but was adopted as a fixed upstream injection configuration based on our previous systematic comparison of three hydrogen-injection strategies, namely co-flow injection, straight-pipe injection, and counter-flow injection [22]. Under identical operating and geometric conditions, the previous study showed that counter-flow injection provided the highest near-field mixing uniformity and the shortest mixing distance.

The enhanced mixing produced by the counter-flow configuration was attributed to the increased relative velocity between the hydrogen stream and the natural-gas main flow, which strengthened reverse shear, local recirculation, and radial species redistribution near the injection region. However, the previous study also showed that counter-flow injection resulted in a higher pressure drop along the hydrogen-supply path. Therefore, this configuration should be regarded as a trade-off between rapid mixing and additional injection-side hydraulic loss, rather than as an intrinsically energy-saving arrangement.

In the present study, the hydrogen-injection configuration was kept fixed, while the contraction angle, throat length, and diffuser angle of the Venturi section were selected as the optimization variables.

Figure 1b presents the detailed geometric design of the Venturi section. The three key geometric parameters selected in this study are the contraction angle, throat length, and diffuser angle. The numerical ranges of all geometric parameters are summarized in Table 1.

### 2.2. Mathematical Method

The numerical model used in this study is described below. The flow of hydrogen and natural gas in the mixing device satisfies the continuity equation [23]:

(1) $$\nabla \cdot \left(\rho u\right)=0$$

where *ρ* is gas density, and *u* is the velocity vector. The momentum conservation equation for the mixing and pipeline-flow system is given by the following:

(2) $$\rho u\cdot \nabla u=-\nabla p+\nabla \cdot \left(\tau \right)+\rho g+F$$

where *p* is static pressure, *g* is the gravitational acceleration vector, F denotes body forces other than gravity, and *τ* is the viscous stress tensor. To account for the turbulent characteristics of the mixing flow, the standard k-ε model was used to close the Reynolds-averaged Navier–Stokes equations. The energy conservation equation can be written as follows:

(3) $$\nabla \cdot [u(\rho E+p)]=\nabla \cdot \left[k_{\mathrm{eff}}\nabla T+\left(\tau_{\mathrm{eff}}\cdot u\right)-\sum_{i}h_{i}j_{i}\right]$$

where *E* is the total energy, *T* is temperature, $k_{eff}$ is the effective thermal conductivity, $\tau_{eff}$ is the effective viscous stress tensor, $h_{i}$ is the specific enthalpy of component *i*, and $j_{i}$ represents the diffusion flux of component *i*. During pipeline flow and mixing, mutual diffusion occurs between hydrogen and natural-gas components. The local mass fraction of each species is obtained by solving the convection-diffusion equation for species *i*:

(4) $$\nabla \cdot \left(\rho uY_{i}\right)=-\nabla \cdot j_{i}+S_{i}$$

The diffusion flux of species *i* induced by concentration and temperature gradients is expressed as follows:

(5) $$j_{i}=\rho D_{i,m}\nabla Y_{i}-D_{T,i}\frac{\nabla T}{T}$$

where $D_{i,m}$ is the mass diffusion coefficient of species *i* in the mixture, and $D_{T,i}$, *i* is the thermal diffusion coefficient.

(6) $$D_{i,m}=\frac{1-X_{i}}{\sum_{j,j\neq i}X_{j}/D_{ij}}$$

where $X_{i}$ is the mole fraction of species *i*, and the gas-mixture temperature dependence can be represented by a constant or polynomial function. These mathematical models provide the basis for the numerical simulations of the physical mixer.

In the present study, the flow is fully developed turbulent flow. Based on previous studies by other researchers [24,25,26], the standard k–ε model is employed to predict the turbulent behavior. The turbulence equations are given as follows:

(7) $$\frac{\partial (\rho ku_{i})}{\partial x_{i}}=\frac{\partial }{\partial x_{j}}\left[\left(\mu +\frac{\mu_{t}}{\sigma_{k}}\right)\frac{\partial k}{\partial x_{j}}\right]+G_{k}+G_{b}-\rho \varepsilon -Y_{M}+S_{k}$$

(8) $$\frac{\partial (\rho \varepsilon u_{i})}{\partial x_{i}}=\frac{\partial }{\partial x_{j}}\left[\left(\mu +\frac{\mu_{t}}{\sigma_{\varepsilon }}\right)\frac{\partial \varepsilon }{\partial x_{j}}\right]+C_{1\varepsilon }\frac{\varepsilon }{k}\left(G_{k}+C_{3\varepsilon }G_{b}\right)-C_{2\varepsilon }\rho \frac{\varepsilon^{2}}{k}+S_{\varepsilon }$$

where *G_K_* is the turbulent kinetic energy generated by the mean velocity gradient; *ε* is the turbulence dissipation rate; *k* is the turbulent kinetic energy; *μ_t_* is the turbulent viscosity; *σ_k_* is the turbulence Prandtl number for *k*; *σ_ε_* is the turbulence Prandtl number for *ε*; *G_b_* is the turbulent kinetic energy caused by buoyancy; *Y_M_* is the contribution of fluctuating expansion to the total dissipation rate in compressible turbulence; $S_{k}$ and $S_{\varepsilon }$ are the source terms in the k and ε transport equations, respectively; and $C_{1\varepsilon }$, $C_{2\varepsilon }$, and $C_{3\varepsilon }$ are model constants.

### 2.3. Boundary Conditions and Monitoring-Section Design

After experimental design and geometric modeling, the 15 models were imported into ANSYS Fluent. The fluid domains were extracted in ANSYS SpaceClaim 24 R1, and the built-in repair tools were used to confirm that no defective surfaces existed. Boundary groups were named consistently, including one wall, one hydrogen inlet, one natural-gas inlet, and one fluid domain, so that similar boundary conditions could be repeatedly applied in different simulation cases.

For the two-inlet configuration, the hydrogen blending ratio was calculated as follows [16]:

(9) $$HBR=\frac{v_{2}d_{2}^{2}}{v_{1}d_{1}^{2}+v_{2}d_{2}^{2}}$$

Here, *HBR* denotes the hydrogen blending ratio. In this study, *v*_1_ and *d*_1_ are the inlet velocity and inner diameter of the natural-gas main pipe, respectively, while *v*_2_ and *d*_2_ are the inlet velocity and inner diameter of the hydrogen pipe. With *v*_1_ = 10 m/s and *v*_2_ = 12.04 m/s, the *HBR* is 7%. The mixer outlet was defined as a pressure outlet. The ambient temperature was set to 300 K. The standard-wall-function k-ε model was used as the turbulence model. Gravity was applied in the negative y-direction with a magnitude of 9.81 m/s^2^. The numerical settings and boundary conditions are summarized in Table 2. The spatial discretization scheme is presented in Table 3.

To comprehensively analyze the internal flow and energy loss of the Venturi-type mixer, monitoring planes and monitoring points were arranged in the computational domain. In the axial direction, monitoring sections were set before the contraction-section inlet and downstream of the diffuser outlet. In Fluent, yz planes were generated along the x-axis. The surface of main stream inlet was set at x = 0 m, and the first monitoring plane was set at x = 0.5 m. The second plane began at x = 1.3 m, and subsequent planes were spaced by 0.1 m. Thus, the 25 monitoring planes extended to x = 3.7 m.

To obtain the mixture distribution at outlet sections and calculate mixing uniformity, multiple layers of monitoring points were arranged on the cross-section. The points were generated programmatically using a radial layered arrangement, with points at different radii alternately placed in the axial and 45° diagonal directions, resulting in 25 sets of monitoring points across the cross-section.

### 2.4. Mesh Generation and Grid-Independence Verification

**Figure 2:**
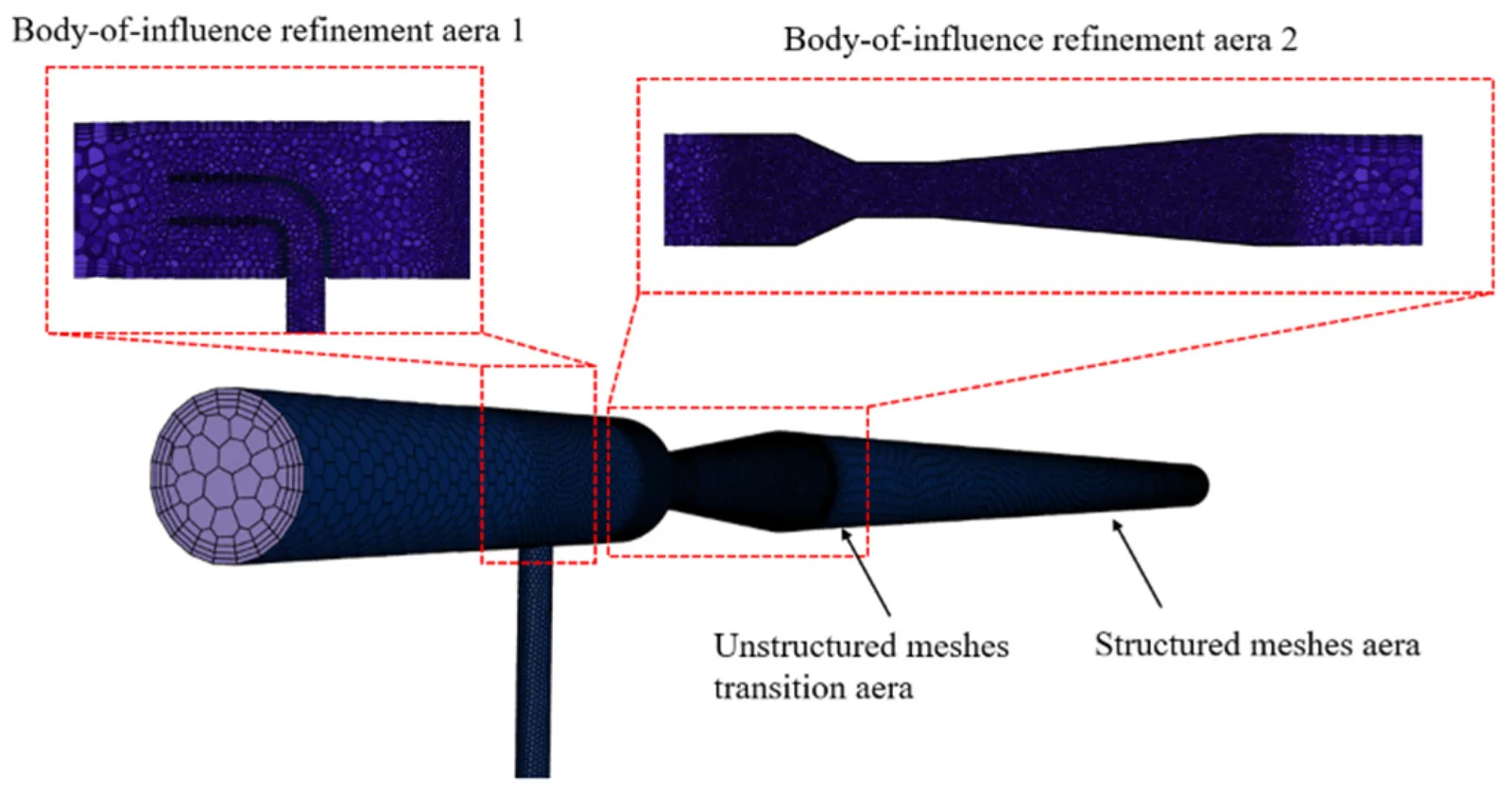
Schematic diagram of local mesh refinement.

Mesh generation is a critical step in numerical simulation because mesh quality directly affects accuracy and convergence. Structured meshes typically provide high quality and low memory consumption but require relatively regular domains. Unstructured meshes offer strong geometric adaptability and flexible node control, although they may produce a large number of cells and require more memory. For the Venturi-type mixer studied here, complex geometry is concentrated near the hydrogen injection section and the Venturi core area, while the remaining regions are relatively regular. Therefore, a hybrid meshing strategy combining structured and unstructured meshes was adopted. Local body-of-influence refinement was applied to the hydrogen injection section and Venturi section, as shown in Figure 2. Notably, although the Venturi-type hydrogen–natural gas mixer proposed in this study is geometrically axisymmetric, a symmetric computational domain was not adopted. Instead, a full three-dimensional computational domain was employed to avoid imposing artificial symmetry constraints on the hydrogen–natural gas mixing process and to better capture the possible three-dimensional evolution of local concentration gradients and flow non-uniformities.

Before formal numerical simulations, a systematic grid-convergence assessment was conducted following the procedure proposed by Celik et al. [27]. Three geometrically similar meshes were generated with the same mesh topology, refinement regions, boundary-layer treatment, and growth rate. The effective grid refinement ratios were evaluated using the representative cell size, as shown in Table 4.

The reference center configuration as our optimization process chosen below, with a contraction angle of 22.7°, a throat length of 70 mm, and a diffuser angle of 6.5°, was selected for the grid-convergence study. All physical models, boundary conditions, spatial discretization schemes, and convergence criteria were kept unchanged. Total-pressure loss, outlet mixing uniformity, and maximum throat velocity were selected as representative quantities because they characterize both the global hydraulic performance and the local mixing process.

The apparent order of convergence, Richardson-extrapolated solution, and Grid Convergence Index were calculated using the three-grid procedure. As shown on Table 5 The adopted-grid GCI values for total-pressure loss and mixing uniformity were 0.27% and 16.21%, respectively. In addition, the asymptotic convergence ratios were 1.01 and 1.03, both close to unity. These results indicate that the numerical solutions are within the asymptotic grid-convergence range.

Although the finest grid provided the lowest discretization uncertainty, the adopted grid produced deviations of 0.27% and 16.21% in pressure loss and mixing uniformity, respectively, while substantially reducing computational cost. Therefore, the medium grid was adopted for the parametric and response-surface simulations.

**Figure 3:**
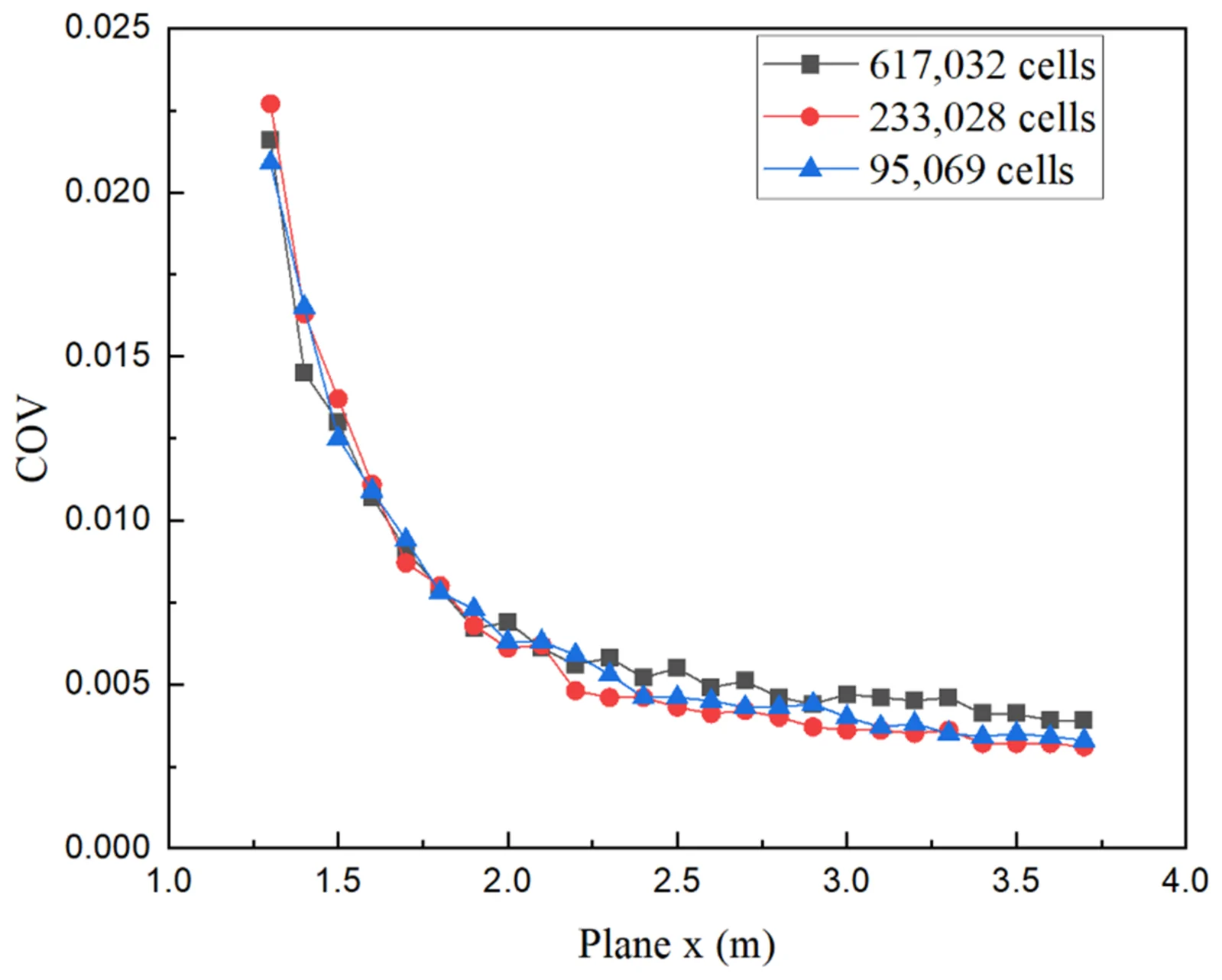
Grid-independence verification.

As shown in Figure 3, the overall trend and errors changed slightly. Considering both accuracy and local computational resources, the adopted mesh 233028 was selected among the stable fine and ultra-fine meshes.

After preprocessing the Venturi premixer models, the three-dimensional models corresponding to the 15 experimental cases were meshed and checked. Local refinement was applied to the contraction section, throat, diffuser, and hydrogen injection inlet, where velocity gradients and turbulence intensity vary sharply. Structured hexahedral meshes were used in the main pipe region to reduce numerical diffusion, whereas unstructured tetrahedral or hybrid meshes were applied in geometrically complex regions such as the throat and diffuser.

### 2.5. Model Validation

Before conducting the main simulations, the numerical model and computational method were validated. The 90° T-pipe blending model established by Tao Di et al. was selected as the reference case [19]. Their study focused on hydrogen blending, adopted the coefficient of variation (COV) as the concentration-uniformity metric, and used axial position to characterize downstream mixing development. The main-pipe inlet, hydrogen inlet, and outlet were specified as velocity inlets and pressure outlet, and COV and pressure drop were the main performance indicators. In this study, the 90° mixing structure was rebuilt in ANSYS Fluent, and the same meshing strategy, species-transport model, and turbulence-solution method as the present main study were applied. Hydrogen concentration distributions on different axial sections were extracted and used to calculate COV values for comparison with the reference results.

**Figure 4:**
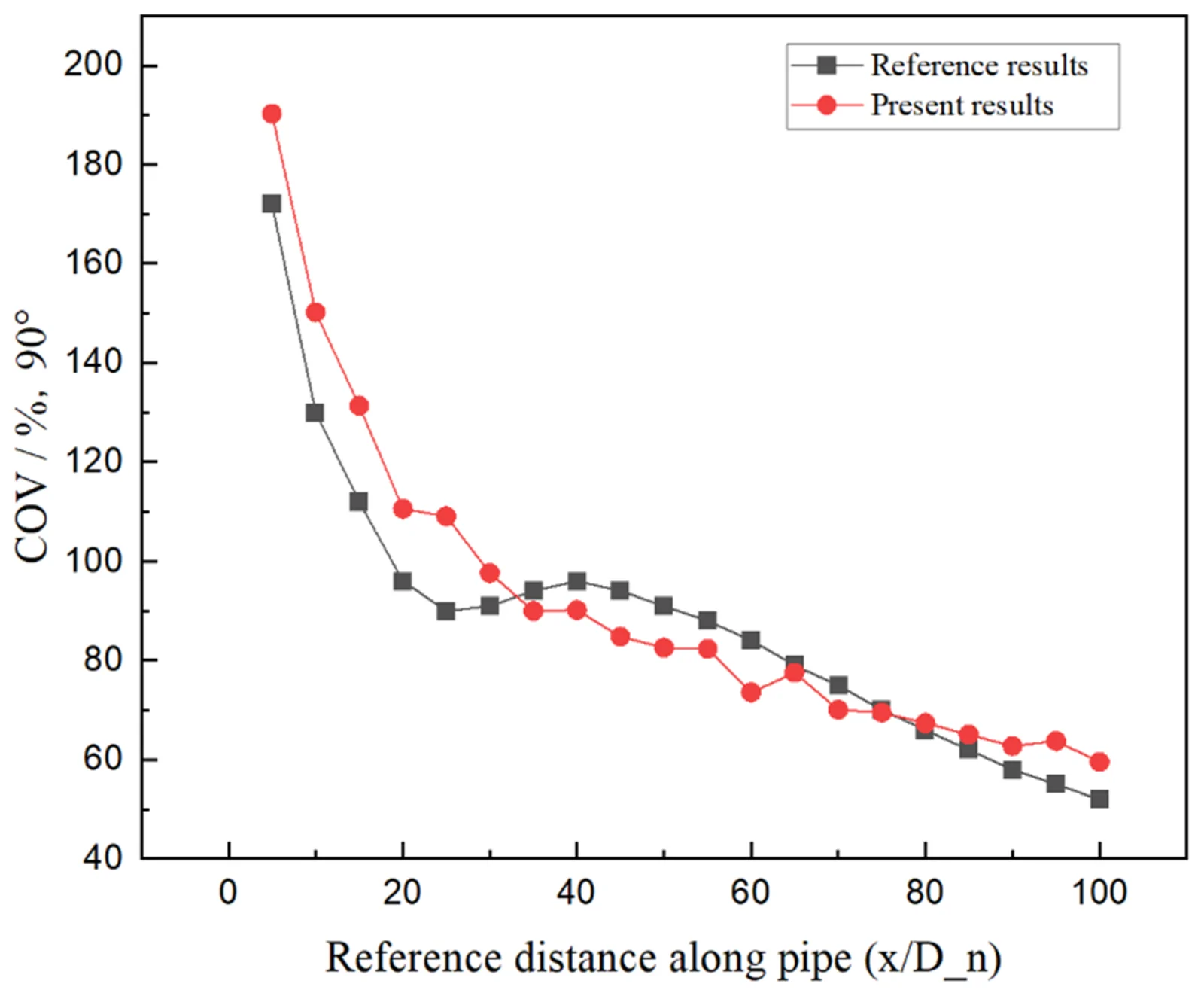
Validation of the present numerical model. The reference data were obtained from [19].

As shown in Figure 4, the present simulation results are consistent with the reference trend. Both curves show a rapid decrease in COV near the inlet, a reduced decreasing rate in the intermediate region, and a slow decline farther downstream. The X/D_n_ follows the original definition of the reference paper and indicates the distance on the x-axis divided by D_n_, the diameter of the main pipe. This indicates that the present model captures the enhanced mixing caused by strong shear, mean vortex development, and turbulent diffusion when the hydrogen jet enters the main pipe. The reference work attributes the rapid near-field decay of COV to the stretching and folding of concentration fields by large-scale coherent vortices, which increase interfacial area and accelerate homogenization. The same physical mechanism is reflected in the present calculation.

Numerically, the present results are slightly higher or lower than the reference values at some sections, but the overall trend agrees well. The differences may arise from turbulence modeling, mesh density, physical-property settings, and discretization schemes. Tao Di et al. [19] used a high-fidelity LES method, whereas the present work uses a RANS turbulence model that is more suitable for engineering parameter sweeps. LES resolves large-scale unsteady vortices more fully, while RANS focuses on the mean flow. Therefore, deviations in the near-inlet high-shear region and downstream mean vortex-decay region are reasonable. Overall, the Fluent modeling method, boundary-condition settings, and post-processing approach used in this study are sufficiently reliable for subsequent numerical analysis of the Venturi-type mixer.

## 3. Optimization Method

### 3.1. Establishment and Simulation of the Response-Surface Model

Response-surface methodology (RSM) is a statistical method for optimizing stochastic or complex processes [28]. It uses polynomial functions to numerically fit the relationship between response indicators and factors, thereby providing quantitative relationships among variables. Because the constructed multidimensional response surface approximates practical behavior while requiring relatively few test cases, RSM has been widely used in fluid-dynamics optimization.

Considering the complexity and coupling among the three variables, the response-surface model is generally expressed as follows:

(10) $$y=\beta_{0}+\sum_{i=1}^{k}\beta_{i}x_{i}+\sum_{i=1}^{k}\beta_{ii}x_{i}^{2}+\sum_{i=1}^{k-1}\sum_{j=i+1}^{k}\beta_{ij}x_{i}x_{j}+\varepsilon$$

where y is the response, *β*_0_ is the constant term, *β_i_* are linear regression coefficients, *β_ii_* are quadratic coefficients, *β_ij_* are interaction coefficients, *x_i_* and *x_j_* are design variables, *k* is the number of design variables, and *ε* is the random error.

Two common experimental designs for RSM are central composite design (CCD) and Box–Behnken design (BBD). BBD can analyze and model the relationship between independent variables and responses through regression equations. Compared with CCD, BBD requires fewer experiments while still fitting a quadratic response surface, thereby improving experimental efficiency under limited resources. It is also effective for identifying interactions among variables [29,30].

**Figure 5:**
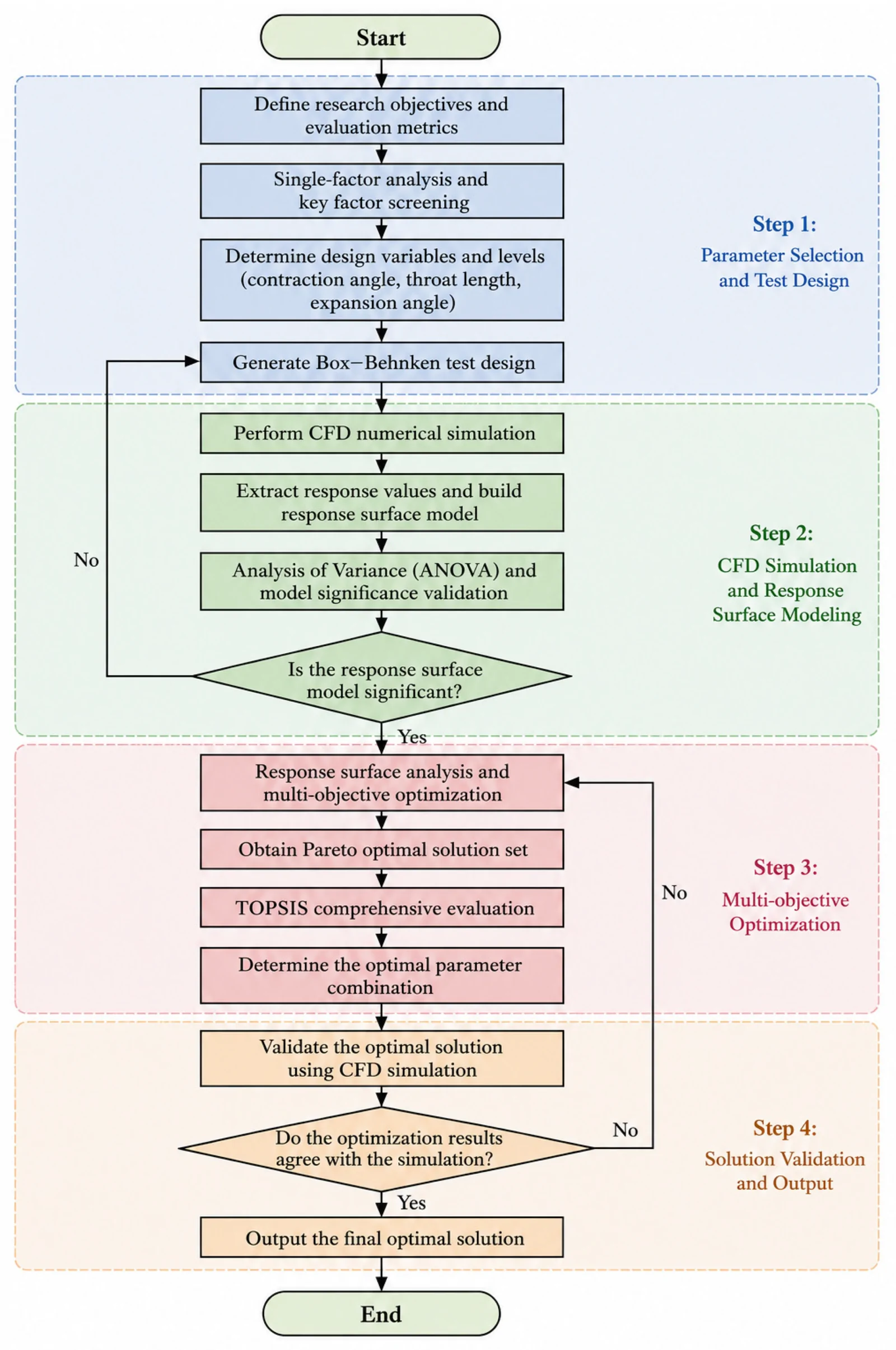
Flowchart of response-surface optimization.

The RSM design process used in this study is shown in Figure 5. Design variables and objective functions were first defined. A BBD matrix was then generated, and numerical results based on the RANS turbulence model were used to construct mathematical response models. Analysis of variance (ANOVA) was used to evaluate model significance. If the model was significant, response-surface analysis was performed; otherwise, the design was adjusted and the procedure repeated. During optimization, predicted optimum results were validated by numerical simulations. If the results agreed, the optimum solution was accepted. If obvious discrepancies appeared, optimization constraints or weight factors were adjusted, and the optimization was repeated.

### 3.2. Optimization Objectives

To systematically and parametrically evaluate the influence of structural parameters on the mixing process, mixing uniformity and energy loss were selected as the two key performance indicators, while TOPSIS was used as the main comprehensive index for verifying the optimization results.

#### 3.2.1. Mixing Uniformity

In the numerical simulations, the hydrogen volume fraction was used as the basic variable describing the mixing state. The outlet section of the mixer was selected for statistical analysis of the hydrogen volume fraction over the section.

First, the area or point-averaged hydrogen volume fraction on the section is defined as follows:

(11) $$\bar{Y}=\frac{1}{N}\sum_{i=1}^{N}Y_{i}$$

where $\bar{Y}$ is the mean hydrogen volume fraction over the section, *N* is the number of sampling points or cells, $Y_{i}$ is the hydrogen volume fraction at sampling point *i*, and *i* is the sampling index.

The standard deviation is then introduced as follows:

(12) $$\sigma =\sqrt{\frac{1}{N-1}\sum_{i=1}^{N}(Y_{i}-\bar{Y}{)}^{2}}$$

where σ is the standard deviation of the hydrogen volume fraction, indicating the concentration fluctuation intensity. The term $Y_{i}-\bar{Y}$ represents the local deviation from the mean, and *N* − 1 is the degrees-of-freedom correction for an unbiased estimate.

The coefficient of variation (COV) [19] is used as the mixing-uniformity metric:

(13) $$COV=\frac{\sigma }{\bar{Y}}$$

where *COV* is a dimensionless relative concentration fluctuation, *σ* is the absolute fluctuation intensity, and $\bar{Y}$ is used for normalization.

For engineering comparison, the mixing uniformity index is further defined as follows:

(14) $$\mu =\left(1-COV\right)\times 100\%$$

When *μ* approaches 100%, hydrogen is nearly uniformly distributed over the section. In engineering applications, a high *μ* value is commonly used as the criterion for complete or near-complete gas mixing, and this metric is widely adopted in static-mixer and fuel-premixer studies [31]. For the optimization process, we chose the COV at plane 13 as target, since it is the outlet surface of the Venturi mixing session.

#### 3.2.2. Energy Loss

In gas mixing, irreversible energy dissipation is caused by contraction, shear, turbulence, and wall friction, and it is mainly reflected by total-pressure loss. Therefore, this study uses total-pressure loss to characterize the energy loss of the mixing process [32,33]. The first monitoring plane at x = 0.5 m and the 25th monitoring plane at x = 3.7 m were selected as reference sections, and their area-averaged total pressures were used to define the total-pressure loss:

(15) $$\Delta P_{t}={\bar{P}}_{t1}-{\bar{P}}_{t2}$$

where $\Delta P_{t}$ is the total-pressure loss in Pa, and ${\bar{P}}_{t1}$ and ${\bar{P}}_{t2}$ are the area-averaged total pressures at the inlet and downstream reference sections, respectively. Together with the COV-based mixing-uniformity index (*μ*), $\Delta P_{t}$ provides a two-dimensional assessment of mixing quality and energy loss.

The COV-based uniformity index is widely used to quantify the spatial non-uniformity of species concentration in static mixers [17,34]. Together with the area-averaged pressure loss, it provides an engineering assessment of the trade-off between mixing quality and energy consumption.

#### 3.2.3. TOPSIS Comprehensive Evaluation Index

Because multi-objective optimization is complex, comparing pressure loss or mixing uniformity alone cannot intuitively identify the best overall scheme [35]. To verify the rationality of the genetic-algorithm optimization, the TOPSIS (Technique for Order Preference by Similarity to Ideal Solution) method was introduced for comprehensive evaluation.

The core idea of TOPSIS is that the optimal alternative should be closest to the positive ideal solution and farthest from the negative ideal solution. Pressure loss and mixing uniformity were selected as the evaluation indicators, with pressure loss treated as a cost-type indicator and mixing uniformity as a benefit-type indicator. After constructing the weighted normalized evaluation matrix, the positive ideal solution and negative ideal solution were defined, and the Euclidean distances from each candidate to these ideal solutions were calculated:

(16) $$D_{i}^{+}=\sqrt{\sum_{j=1}^{n}{\left(v_{ij}-A_{j}^{+}\right)}^{2}}$$

(17) $$D_{i}^{-}=\sqrt{\sum_{j=1}^{n}{\left(v_{ij}-A_{j}^{-}\right)}^{2}}$$

where $D_{i}^{+}$ and $D_{i}^{-}$ are the distances from scheme *i* to the positive and negative ideal solutions, respectively, and $v_{ij}$ is the weighted normalized value of scheme *i* for indicator *j*. The relative closeness of each scheme is then calculated as follows:

(18) $$C_{i}=\frac{D_{i}^{-}}{D_{i}^{+}+D_{i}^{-}}$$

where $C_{i}$ is the relative closeness of scheme *i*. $C_{i}$ ranges from 0 to 1. A value closer to 1 indicates a scheme closer to the positive ideal solution and therefore better comprehensive performance; a value closer to 0 indicates poorer performance.

## 4. Results and Discussion

### 4.1. Single-Factor Analysis

Based on the response-surface experiments, single-factor analysis was conducted to further clarify the independent effects of the main geometric parameters of the Venturi-type mixer. The contraction angle, throat length, and diffuser angle were varied separately. The center-point case of the response-surface design, with a contraction angle of 22.7°, throat length of 70 mm, and diffuser angle of 6.5°, was used as the reference for single-factor variation.

#### 4.1.1. Effect of Contraction Angle

The contraction angle is an important geometric parameter of the contraction section. It determines the acceleration intensity of the main flow before entering the throat and affects the formation of the low-pressure region. As shown on Table 6, five contraction-angle levels were considered: 18.7°, 20.7°, 22.7°, 24.7°, and 26.7°. The throat length was fixed at 70 mm, and the diffuser angle was fixed at 6.5°. Other boundary conditions were identical to the reference central-point structure case.

**Figure 6:**
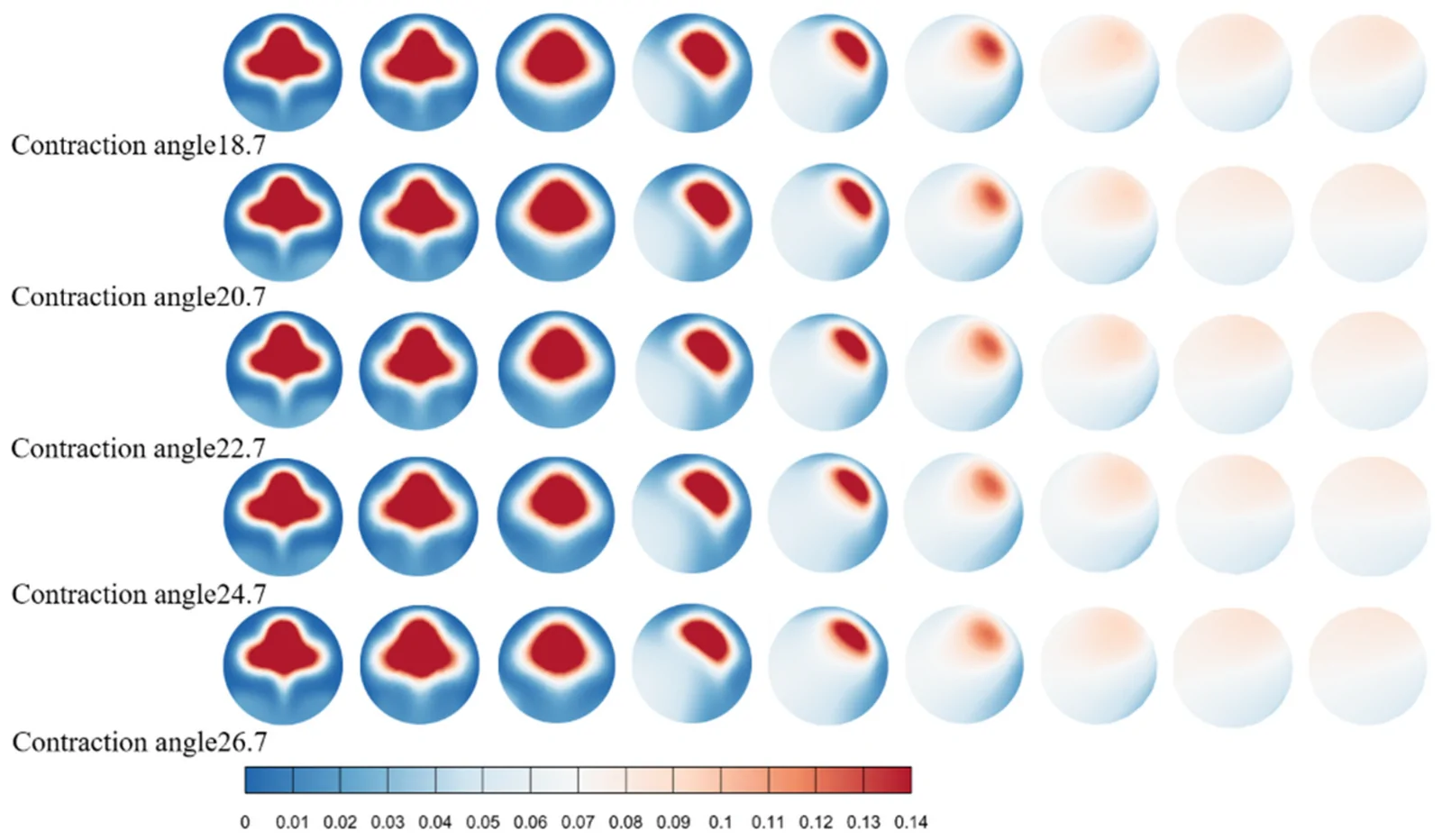
Hydrogen mole-fraction sections for different contraction angles.

**Figure 7:**
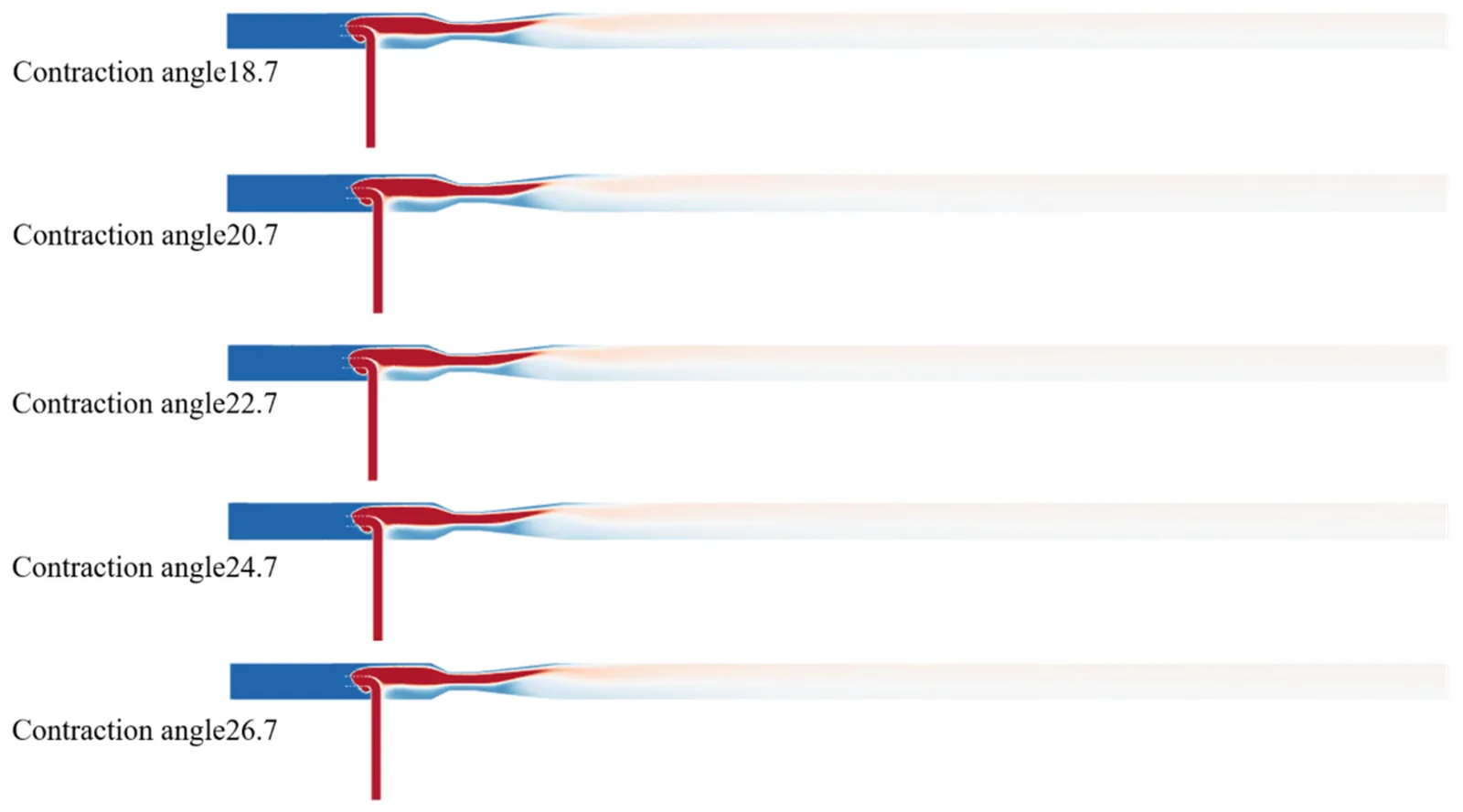
Longitudinal hydrogen mole-fraction distributions for different contraction angles. The color scale is identical to that in Figure 6, ranging from 0 (blue) to 0.14 (red).

As shown in Figure 6 and Figure 7, when only the contraction angle is changed, the first several cross-sections in all cases exhibit a high-concentration hydrogen core. Hydrogen is mainly concentrated in the upper or eccentric region of the pipe, indicating that the early mixing process is still controlled by the branch jet. As the flow develops downstream, the high-concentration region is stretched by the main flow and migrates diagonally. This indicates that the lateral jet generates a distinct transverse momentum component, and the Venturi contraction further amplifies this asymmetric disturbance.

From the longitudinal sections, a smaller contraction angle produces a gentler acceleration process and a smaller velocity gradient between the hydrogen jet and the natural-gas main flow. The shear layer is insufficiently developed, and the hydrogen-rich region remains relatively continuous near the throat. As the contraction angle increases, the acceleration in the contraction section strengthens; the shear near the throat becomes more pronounced; and the hydrogen jet is compressed, stretched, and deflected more strongly. In cross-sections, this appears as a stronger lateral shift of the high-concentration zone and more significant diagonal transport downstream.

**Figure 8:**
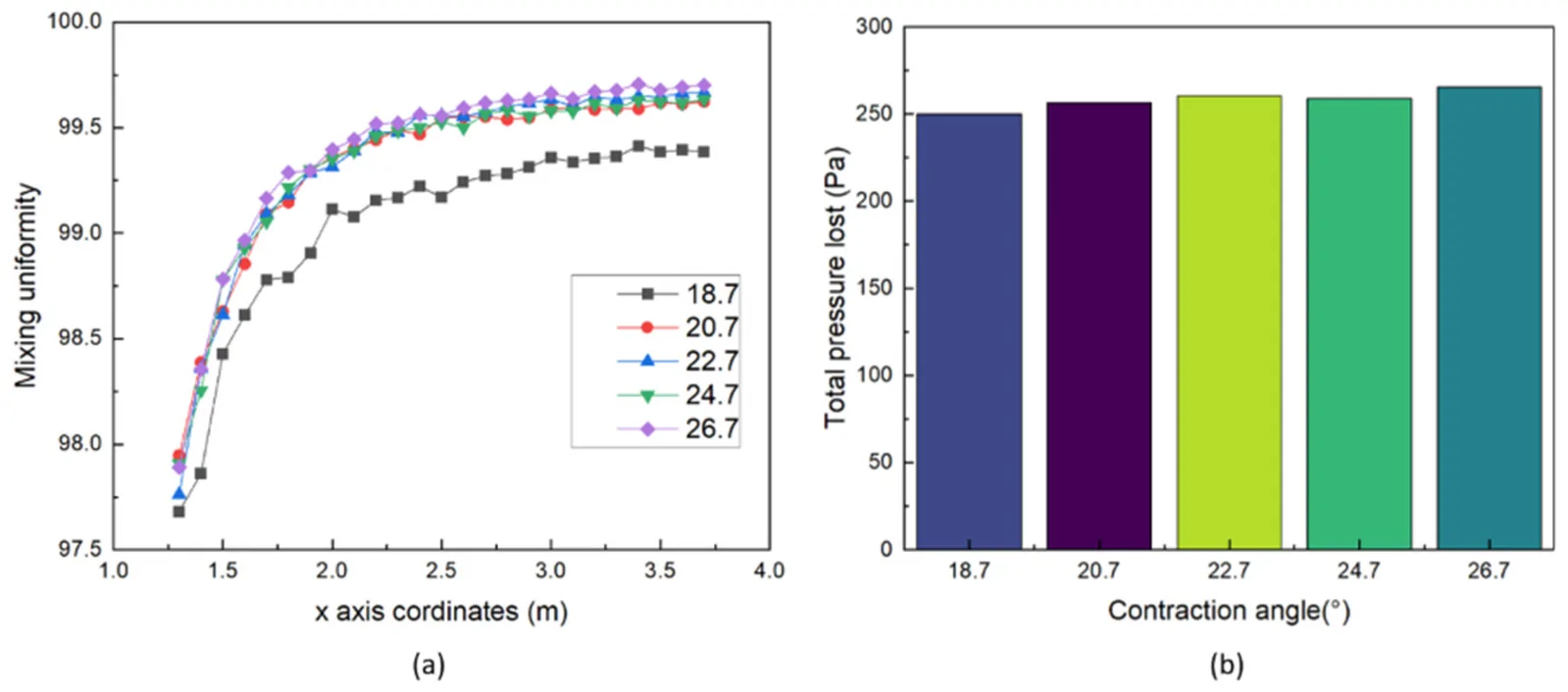
Effects of contraction angle on mixing performance: (**a**) axial distributions of mixing uniformity at different contraction angles; (**b**) total pressure loss at different contraction angles.

Figure 8a shows that differences among contraction angles are mainly reflected in the early mixing rate and final plateau value. The case with an 18.7° contraction angle has the lowest overall mixing uniformity, particularly in the 1.3–2.2 m range. This indicates that a too-small contraction angle provides insufficient main-flow acceleration, weak throat velocity gradients, and limited stretching and breakup of the hydrogen jet. When the contraction angle is increased to 20.7°, 22.7°, 24.7°, or 26.7°, the curves shift upward and the downstream uniformity exceeds 99.5%. Properly increasing the contraction angle enhances the main-flow acceleration and strengthens shear-layer formation near the throat, promoting momentum exchange and species diffusion.

However, the pressure-loss bar chart shows that total-pressure loss increases slowly as the contraction angle increases. The 18.7° case has the lowest pressure loss, while the 26.7° case has the highest. This means that increasing the contraction angle strengthens local acceleration but also increases local resistance and irreversible dissipation. Notably, after the angle increases from 20.7° to 26.7°, the improvement in mixing uniformity becomes small, whereas pressure loss continues to increase. Therefore, the marginal benefit of increasing contraction angle is limited compared with the other factors.

#### 4.1.2. Effect of Throat Length

The throat length determines the residence time of the fluid in the minimum-area region. It directly affects the contact time between the main flow and hydrogen jet in the high-speed shear zone and also influences frictional loss. As shown on Table 7, five throat lengths were considered: 50, 60, 70, 80, and 90 mm. The contraction angle was fixed at 22.7°, and the diffuser angle was fixed at 6.5°.

**Figure 9:**
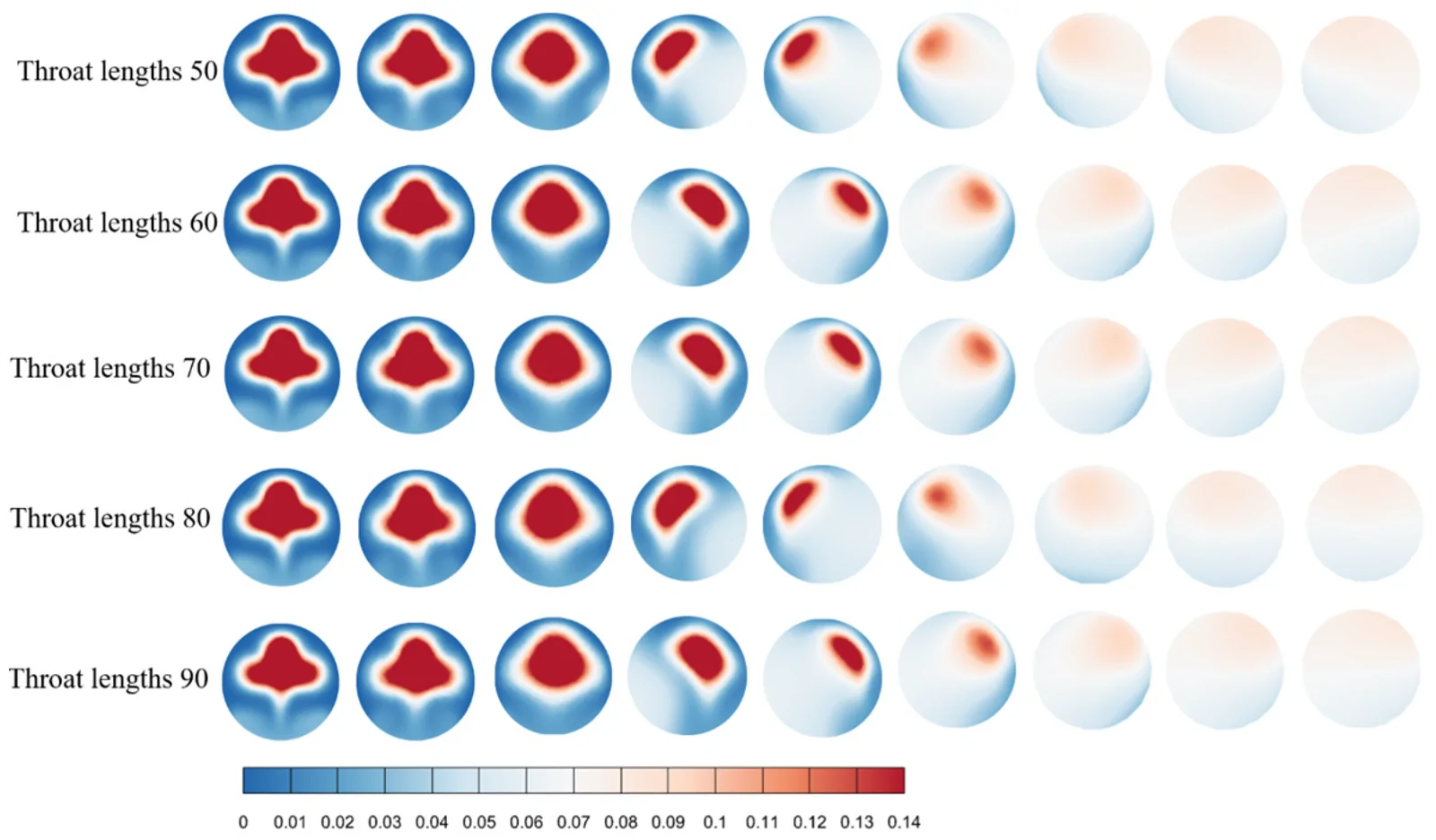
Hydrogen mole-fraction sections for different throat lengths.

**Figure 10:**
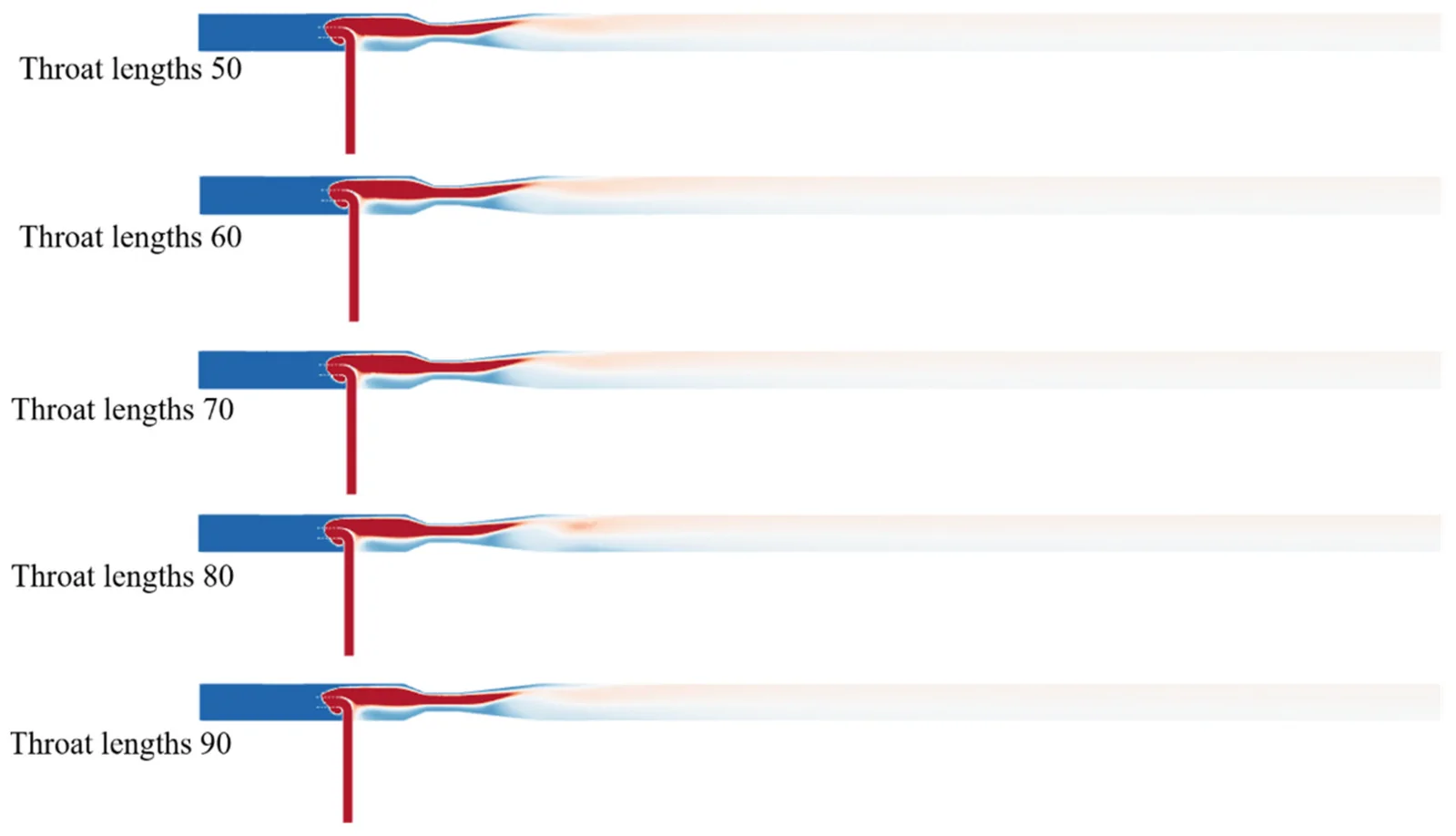
Longitudinal hydrogen mole-fraction distributions for different throat lengths. The color scale is identical to that in Figure 9, ranging from 0 (blue) to 0.14 (red).

When only the throat length changes, Figure 9 and Figure 10 show that the hydrogen-rich region is still formed mainly in the upstream sections and gradually diffuses to the rest of the pipe section downstream. Compared with the contraction angle, throat length has a more obvious effect on the duration of the high-concentration core and its axial stretching.

For a short throat, hydrogen and natural gas have insufficient interaction time in the high-speed throat region. Although the hydrogen jet is affected by main-flow acceleration and shear, the shear layer enters the diffuser before fully developing. Thus, the high-concentration region remains relatively concentrated in cross-sections, and the longitudinal section shows that downstream mixing relies heavily on the subsequent development section.

When the throat length increases to a moderate range, hydrogen resides longer in the high-speed region. The shear between the main and secondary flows can develop continuously, and the hydrogen jet is further stretched and broken. The high-concentration core shrinks faster in cross-sections, and circumferential and radial diffusion become more sufficient.

**Figure 11:**
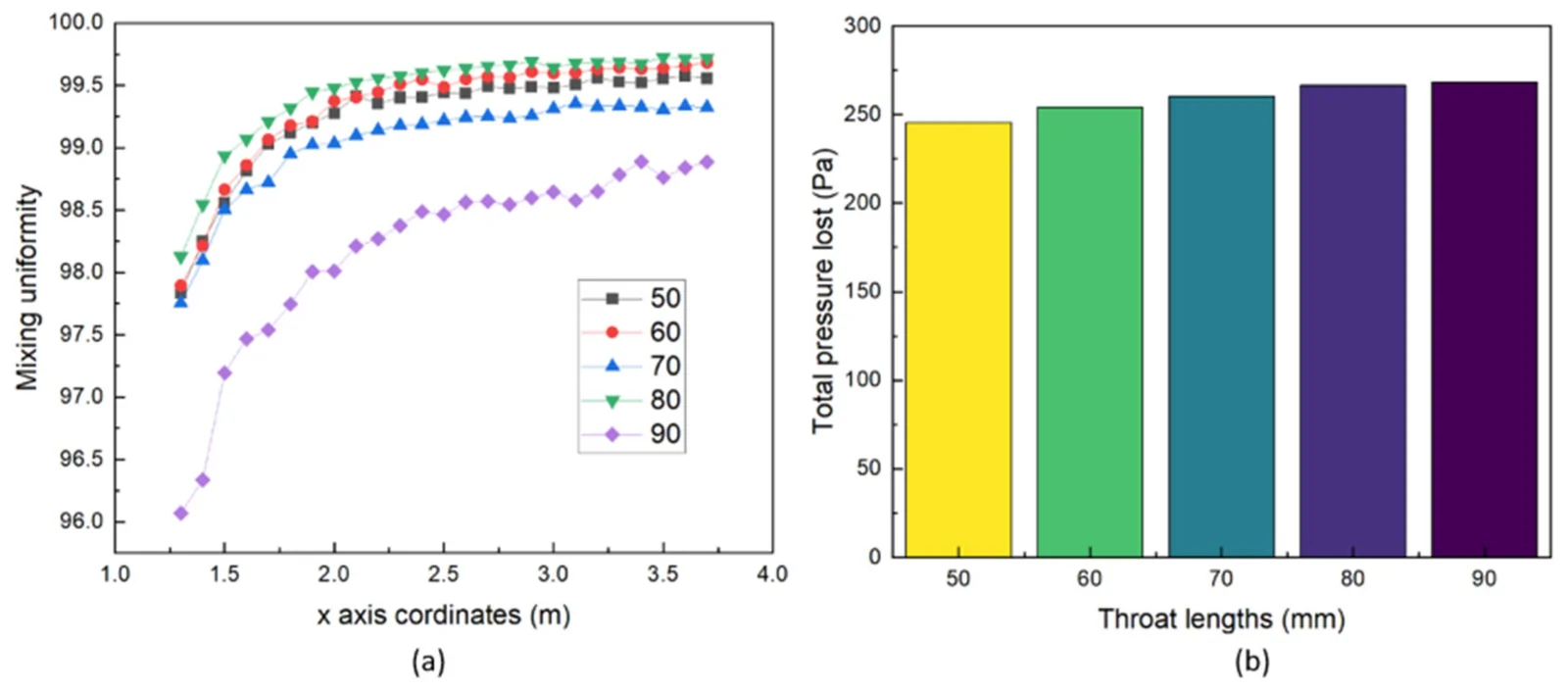
Effect of throat length on mixing performance: (**a**) axial distributions of mixing uniformity at different throat length; (**b**) total pressure loss at different throat length.

However, with an excessively long throat, the high-speed core maintains strong axial stability, and the hydrogen-rich region forms a longer continuous band. A longer throat also increases wall friction loss, making the trade-off between mixing enhancement and energy loss more pronounced. Figure 11 indicates that throat length affects mixing uniformity more significantly than contraction angle. The 50–80 mm cases all improve rapidly downstream and eventually approach or exceed 99.4%. The 80 mm case has the highest curve overall, but the 90 mm case performs abnormally: its near-field uniformity is the lowest and remains below the other cases downstream. Pressure loss increases with throat length because both wall-friction path length and viscous/turbulent dissipation increase. Overall, throat length has a dual effect: a proper throat length enhances mixing by extending shear interaction, while an overly long throat maintains axial stratification and increases pressure loss.

#### 4.1.3. Effect of Diffuser Angle

The diffuser angle is a key parameter governing downstream flow recovery and cross-sectional homogenization. It determines the deceleration rate, pressure recovery, and possibility of boundary-layer separation after the throat flow enters the diffuser. As shown on Table 8, five diffuser-angle levels were considered: 4°, 5°, 6.5°, 8°, and 9°. The contraction angle and throat length were fixed at 22.7° and 70 mm, respectively.

**Figure 12:**
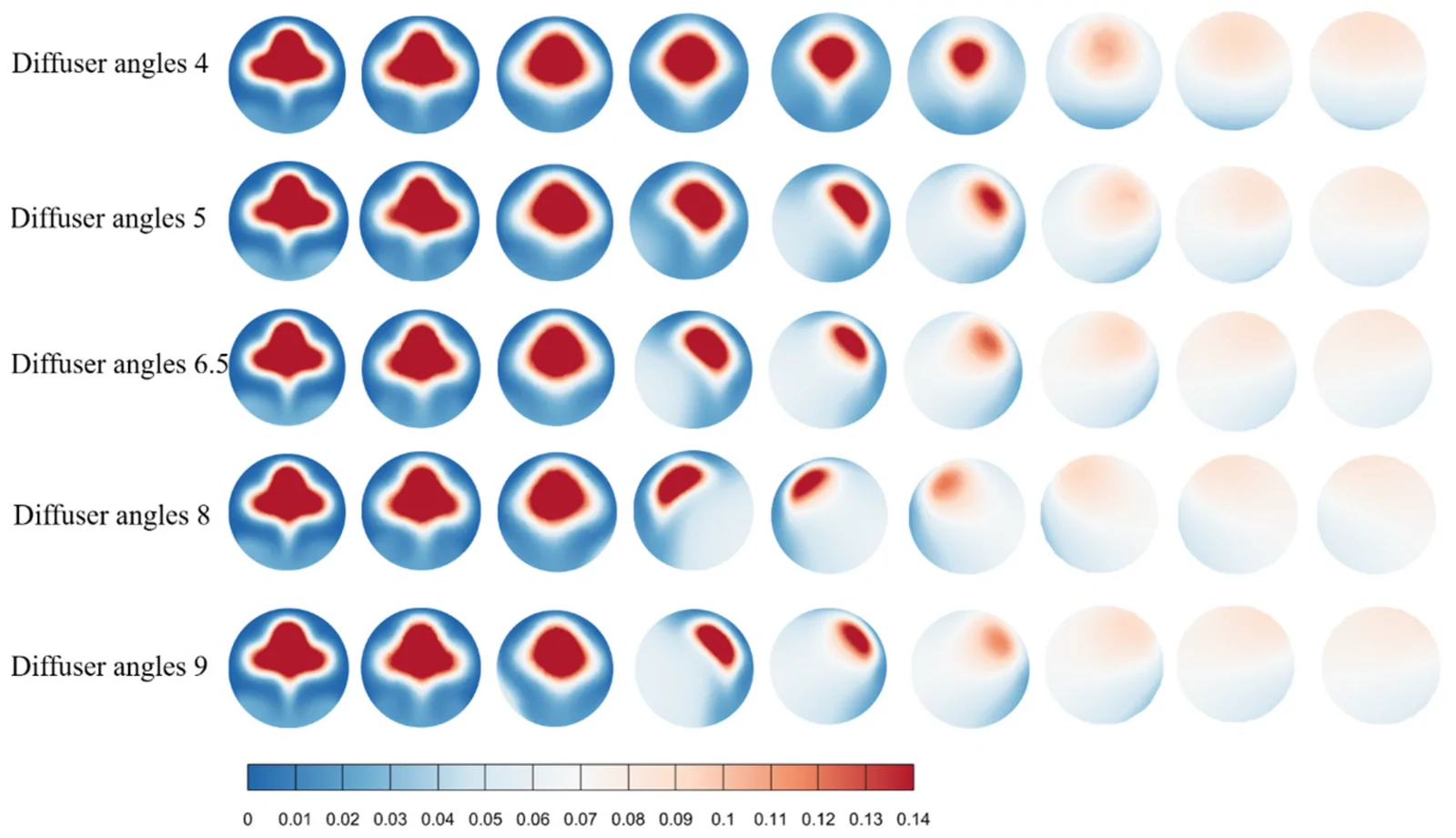
Hydrogen mole-fraction sections for different diffuser angles.

**Figure 13:**
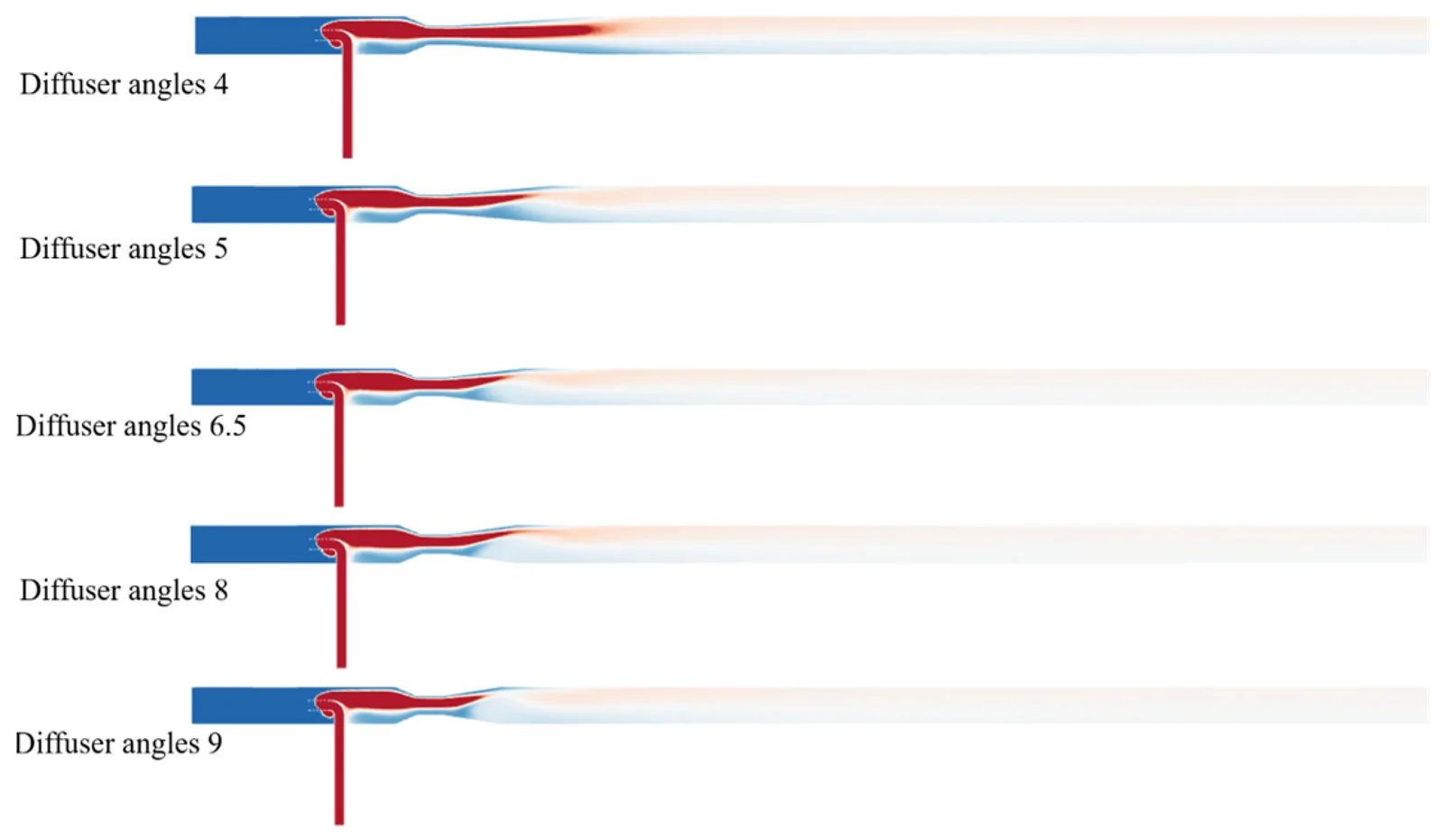
Longitudinal hydrogen mole-fraction distributions for different diffuser angles. The color scale is identical to that in Figure 12, ranging from 0 (blue) to 0.14 (red).

Among the three structural parameters, the diffuser angle has the most significant influence on the mixing process. Figure 12 and Figure 13 show that when the diffuser angle is small, the hydrogen-rich core remains continuous in multiple downstream sections and the high-concentration region decays slowly. This indicates insufficient radial expansion in the diffuser and weak transverse redistribution, especially at 4°, where hydrogen is transported primarily along the pipe axis.

At a moderate diffuser angle, the fluid downstream of the throat undergoes appropriate deceleration and pressure recovery. The adverse pressure gradient can induce moderate recirculation, secondary flow, and sectional rotational motion without causing severe separation. The high-concentration hydrogen region is stretched, deflected, and weakened more rapidly, and the downstream distribution becomes more uniform. Therefore, a moderate diffuser angle favors effective transverse redistribution.

When the diffuser angle is further increased, the adverse pressure gradient in the diffuser becomes stronger, and the tendency for local recirculation and boundary-layer separation increases. Hydrogen may be trapped in local vortical regions, producing local enrichment or eccentric transport. The high-concentration region shifts more clearly in cross-sections, and local rich cores remain in some sections. Consequently, the diffuser angle directly controls pressure-recovery rate, boundary-layer state, secondary-flow intensity, and local recirculation, making it more influential than contraction angle and throat length for final homogenization.

**Figure 14:**
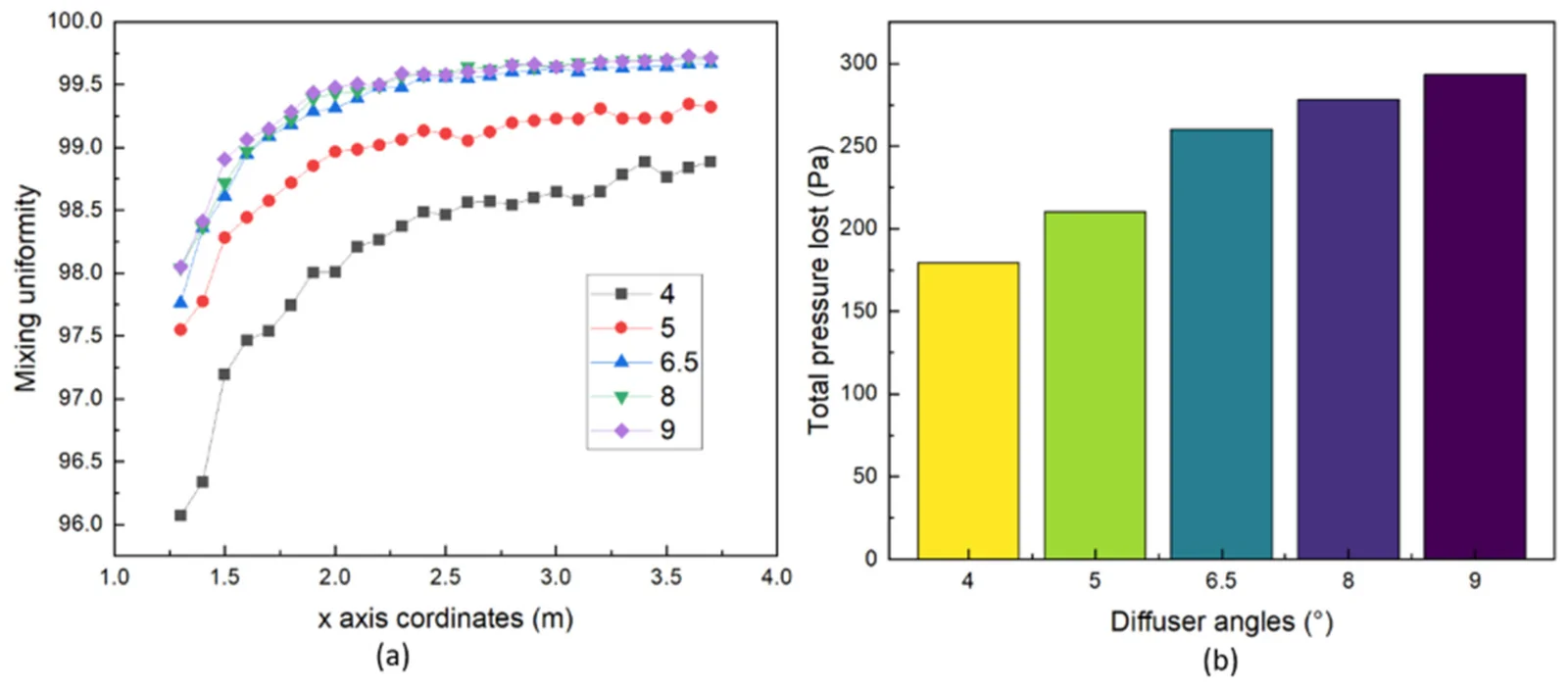
Effect of diffuser angle on mixing performance: (**a**) axial distributions of mixing uniformity at different diffuser angle; (**b**) total pressure loss at different diffuser angle.

Figure 14 further reveals the conflict between mixing enhancement and pressure loss. At 4°, the mixing effect is the weakest because the diffuser lacks radial expansion capability and generates weak secondary flow. Increasing the angle to 5° improves uniformity, while 6.5°, 8°, and 9° produce the highest uniformity, particularly between 1.5 and 2.2 m, and stabilize at approximately 99.6% downstream. However, total-pressure loss increases markedly with diffuser angle: about 180 Pa at 4°, 210 Pa at 5°, 260 Pa at 6.5°, and 280–295 Pa at 8–9°. This sensitivity is much stronger than that of the contraction angle or throat length, indicating that diffuser angle is the most critical parameter controlling energy loss.

### 4.2. Multi-Objective Optimization Analysis

#### 4.2.1. Experimental-Results Analysis

Based on theoretical analysis and related references, the contraction angle, throat length, and diffuser angle were selected as the key structural parameters of the mixer [36]. Each parameter was assigned three levels to ensure both coverage and feasibility, as shown in Table 9.

The complete Box–Behnken matrix and the corresponding responses are listed in Table 10.

As shown in Table 11, ANOVA was used to evaluate the statistical significance of the models, variables, and interactions. The F-value is the ratio of factor mean square to residual mean square and quantifies the relative significance of each term [37]. A larger F-value indicates a more significant effect. The *p*-value evaluates statistical significance, and *p* < 0.05 is generally considered significant [38]. For the COV model, the F-value is 22.54, and *p* = 0.0016; for the Δp model, the F-value is 526, and *p* < 0.0001. These results indicate that both models are highly significant and that the quadratic polynomial model is suitable for predicting pressure loss and its relationship with structural parameters [30].

**Figure 15:**
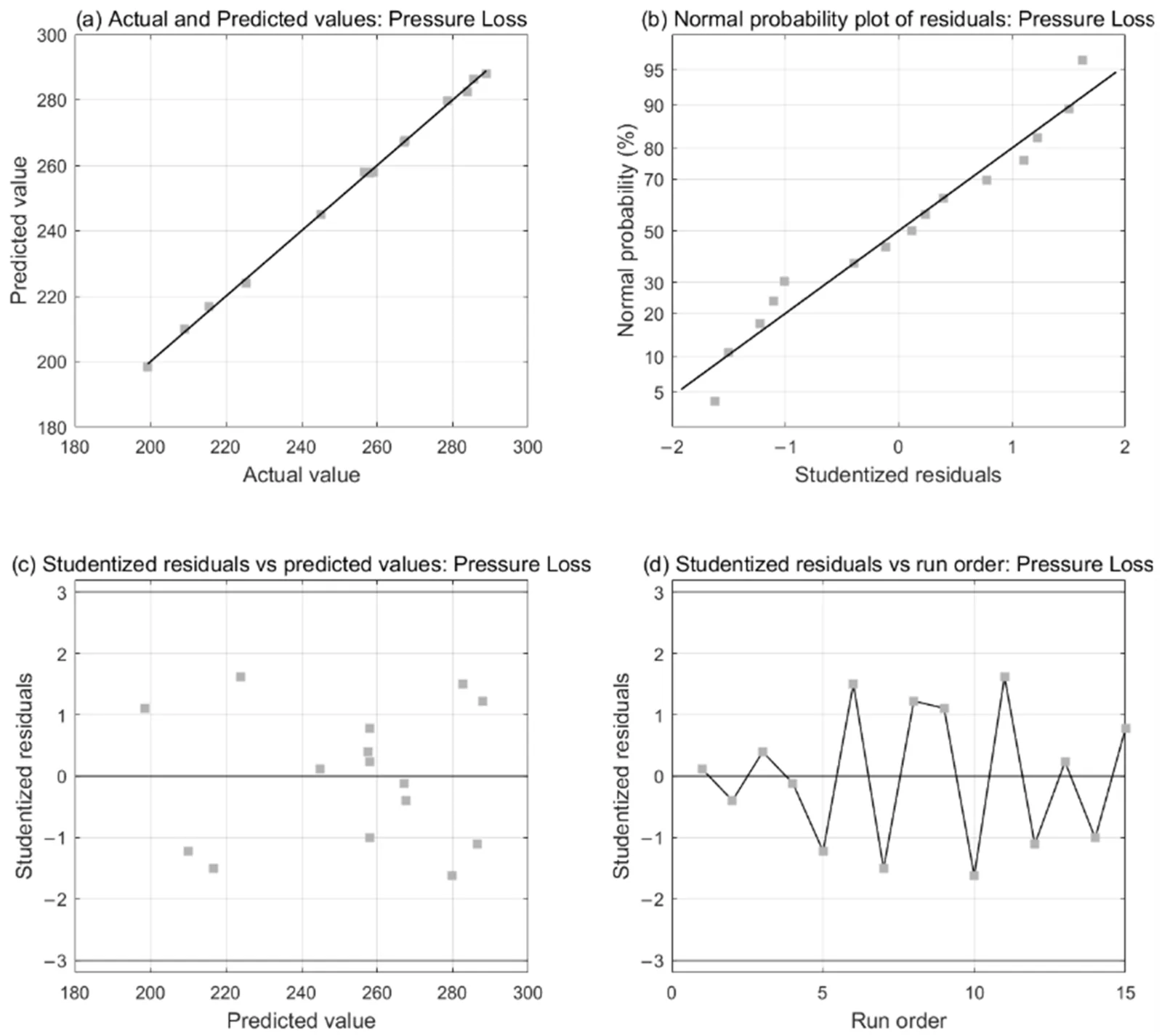
Diagnostic plots for the pressure-loss response model: (**a**) predicted versus CFD-obtained values; (**b**) normal probability plot of studentized residuals; (**c**) studentized residuals versus predicted values; and (**d**) studentized residuals versus run order. Squares denote individual Box–Behnken design runs. The lines represent perfect agreement in (**a**), the fitted normal reference in (**b**), and zero residual and the ±3 diagnostic limits in (**c**,**d**); the polyline in (**d**) connects successive runs.

**Figure 16:**
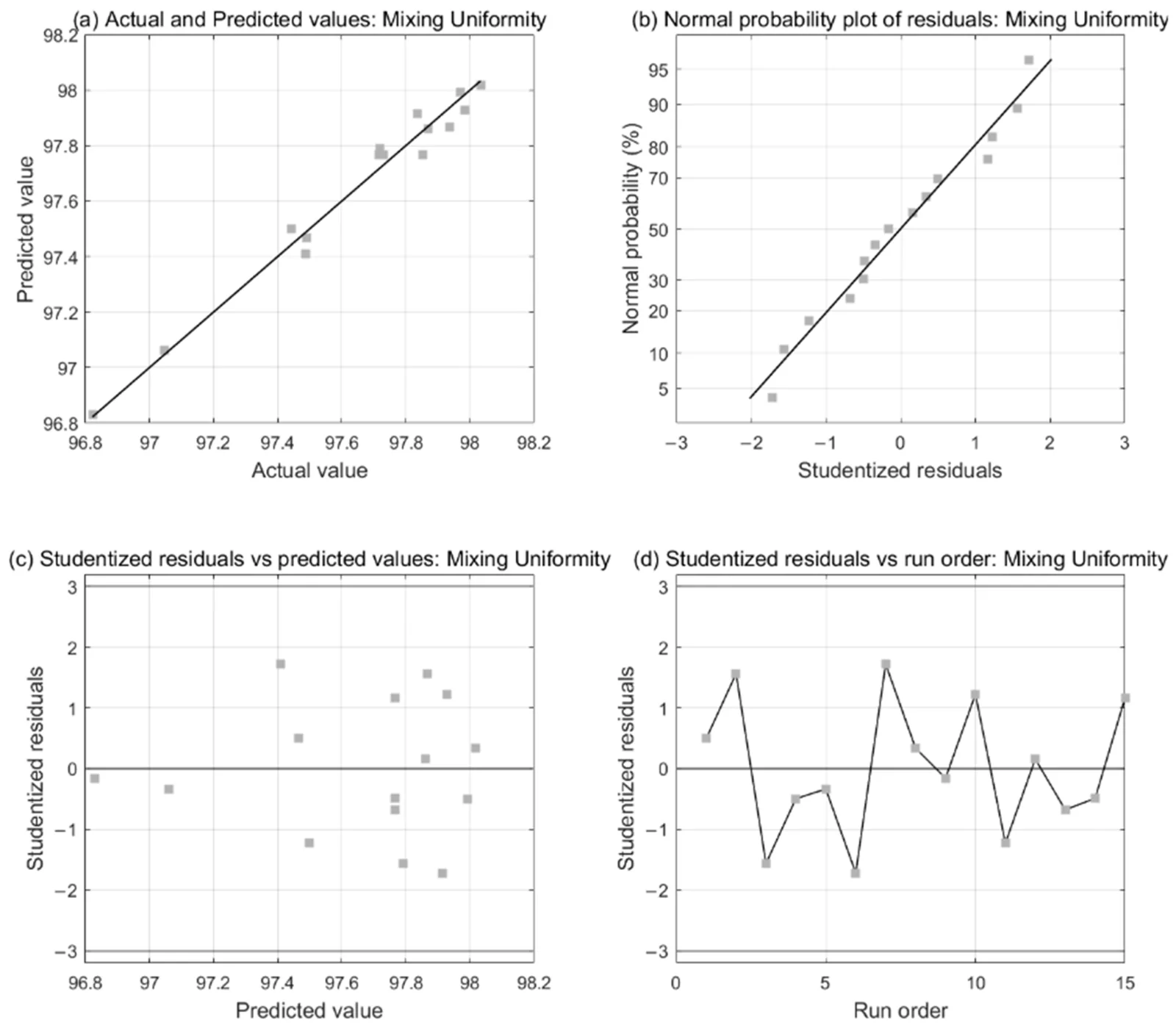
Diagnostic plots for the mixing-uniformity model: (**a**) predicted versus CFD-obtained values; (**b**) normal probability plot of studentized residuals; (**c**) studentized residuals versus predicted values; and (**d**) studentized residuals versus run order. Squares denote individual Box–Behnken design runs. The lines represent perfect agreement in (**a**), the fitted normal reference in (**b**), and zero residual and the ±3 diagnostic limits in (**c**,**d**); the polyline in (**d**) connects successive runs.

To evaluate model adequacy, Figure 15a–d and Figure 16a–d present the actual-versus-predicted plots, normal probability plots of residuals, studentized residuals versus predicted values, and studentized residuals versus scheme number for the pressure-loss and mixing-uniformity models, respectively. The data points in the actual-versus-predicted plots are close to the 45° line, showing good predictive accuracy. The residuals approximately follow the reference line in the normal probability plots, indicating approximate normality. Studentized residuals are randomly distributed around the zero baseline without obvious patterns, indicating that residuals are independent and uncorrelated with predicted values or scheme number. Therefore, the BBD models are adequate and reliable [39].

The structural optimization of the Venturi-type mixer is essentially a multi-objective problem: pressure loss should be minimized, while mixing uniformity remains high. Because pressure loss and mixing uniformity differ in dimension, range, and sensitivity, direct weighting may introduce subjective bias. Therefore, entropy weighting was adopted to assign objective weights to pressure loss and mixing uniformity, and a comprehensive objective function was constructed. A genetic algorithm was then used to search for the optimal structural-parameter combination with low pressure loss and high mixing performance.

Pressure loss was treated as a cost-type indicator, and mixing uniformity as a benefit-type indicator. After nondimensionalization, the information entropy and weights were calculated as follows:

(19) $$e_{j}=-k\sum_{i=1}^{m}p_{ij}\ln p_{ij},{\;p}_{ij}=\frac{y_{ij}}{\sum_{i=1}^{m}y_{ij}},k=\frac{1}{\ln m}$$

(20) $$w_{j}=\frac{1-e_{j}}{\sum_{r=1}^{n}1-e_{r}}$$

where $y_{ij}$ is the normalized dimensionless value of scheme *i* under indicator *j*, $p_{ij}$ is the proportion of scheme *i* under indicator *j*, $e_{j}$ is the information entropy of indicator *j*, $w_{j}$ is the corresponding weight, *m* is the number of samples, and *n* is the number of evaluation indicators. Based on the 15 samples in this study, the weights of pressure loss and mixing uniformity were obtained as follows:

(21) $$w_{\Delta P}=0.74,w_{\mu }=0.26$$

The results indicate that pressure loss has greater dispersion and discrimination than mixing uniformity within the present design range; therefore, it should occupy a larger weight in subsequent optimization and comprehensive evaluation. The response-surface models of pressure loss and mixing uniformity were then incorporated into the comprehensive fitness function, and the coded variables for contraction angle, throat length, and diffuser angle were searched within the prescribed design range.

The fitness function can be expressed as follows:

(22) $$S=w_{\Delta P}\tilde{\Delta P}+w_{\mu }\tilde{\mu }$$

To avoid confusion with previous variables, the normalized variables used in the optimization are denoted as $\tilde{\Delta P}$ and $\tilde{\mu }$ for the normalized pressure loss and mixing uniformity, respectively. Since pressure loss is a cost-type indicator and mixing uniformity is a benefit-type indicator, the two responses were converted into positive dimensionless indicators using min–max normalization:

(23) $$\tilde{\Delta P}=\frac{\Delta P_{max}-\Delta P}{\Delta P_{max}-\Delta P_{min}}$$

(24) $$\tilde{\mu }=\frac{\mu -\mu_{min}}{\mu_{max}-\mu_{min}}$$

The genetic algorithm used a population size of 100, a maximum of 200 generations, and an adaptive feasible mutation strategy. The optimal combination of encoding variables was obtained through iterative search. The values are contraction angle, 20.7°; throat length, 60 mm; and diffusion angle, 5°. This is noted as scheme 16 as shown on Table 10 since it is not in the original 15 scheme of the response-surface model. It should be noted that the optimized solution is located at the lower boundary of the prescribed RSM design space. Therefore, it should be interpreted as the best compromise under the current geometric and engineering constraints rather than a mathematically global optimum. The lower bounds were selected considering manufacturability, sufficient mixing length, and avoidance of excessively weak transverse redistribution, based on the result of our single-factor analysis.

#### 4.2.2. Interaction Effects of Parameters

**Figure 17:**
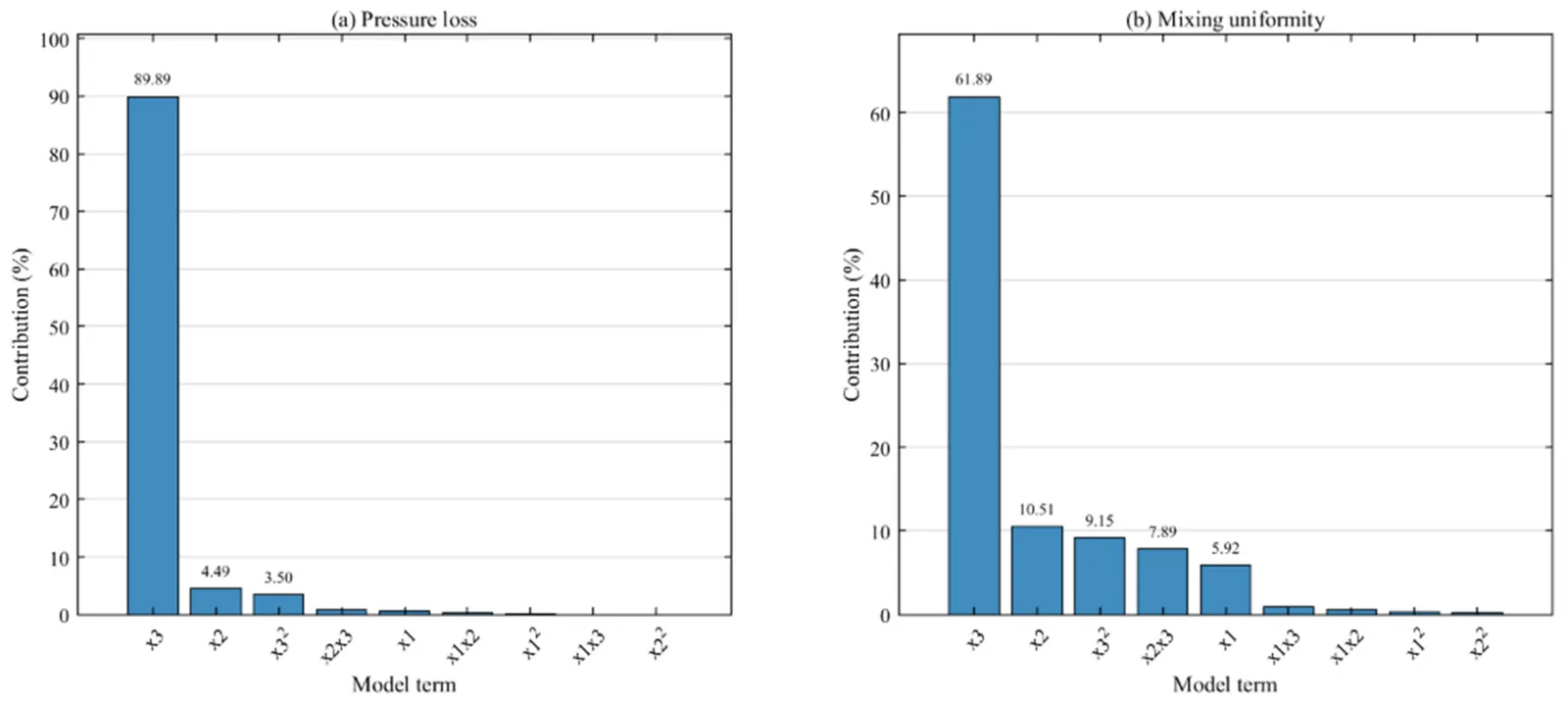
Ranked contributions of the response-surface model terms to (**a**) pressure loss and (**b**) mixing uniformity. Terms are arranged in descending order of contribution.

According to the response-surface model in Equation (10), the coded variables *x*1, *x*2, and *x*3 represent the contraction angle, throat length, and diffuser angle as shown in Figure 17, respectively. To quantify each term’s contribution to the response, the change in model error after deleting a given term was used as an importance measure:

(25) $$C_{i}=\frac{SSE_{reduced,i}-SSE_{full}}{SST}\times 100\%$$

where $C_{i}$ is the contribution rate of term *i*, $SSE_{reduced,i}$ is the residual sum of squares after deleting term *i*, $SSE_{full}$ is the residual sum of squares of the complete quadratic model, and *SST* is the total sum of squares. Figure 17 presents the contribution results after excluding terms with negligible effects.

Figure 17 shows that ×3 has the strongest effect on both mixing uniformity and pressure loss, with contributions of approximately 90% and 60%, respectively, which is consistent with previous conclusions [40]. The contributions of ×2 and the quadratic terms are generally smaller, but the contraction angle, throat length, and certain interaction terms still produce non-negligible effects on mixing uniformity.

**Figure 18:**
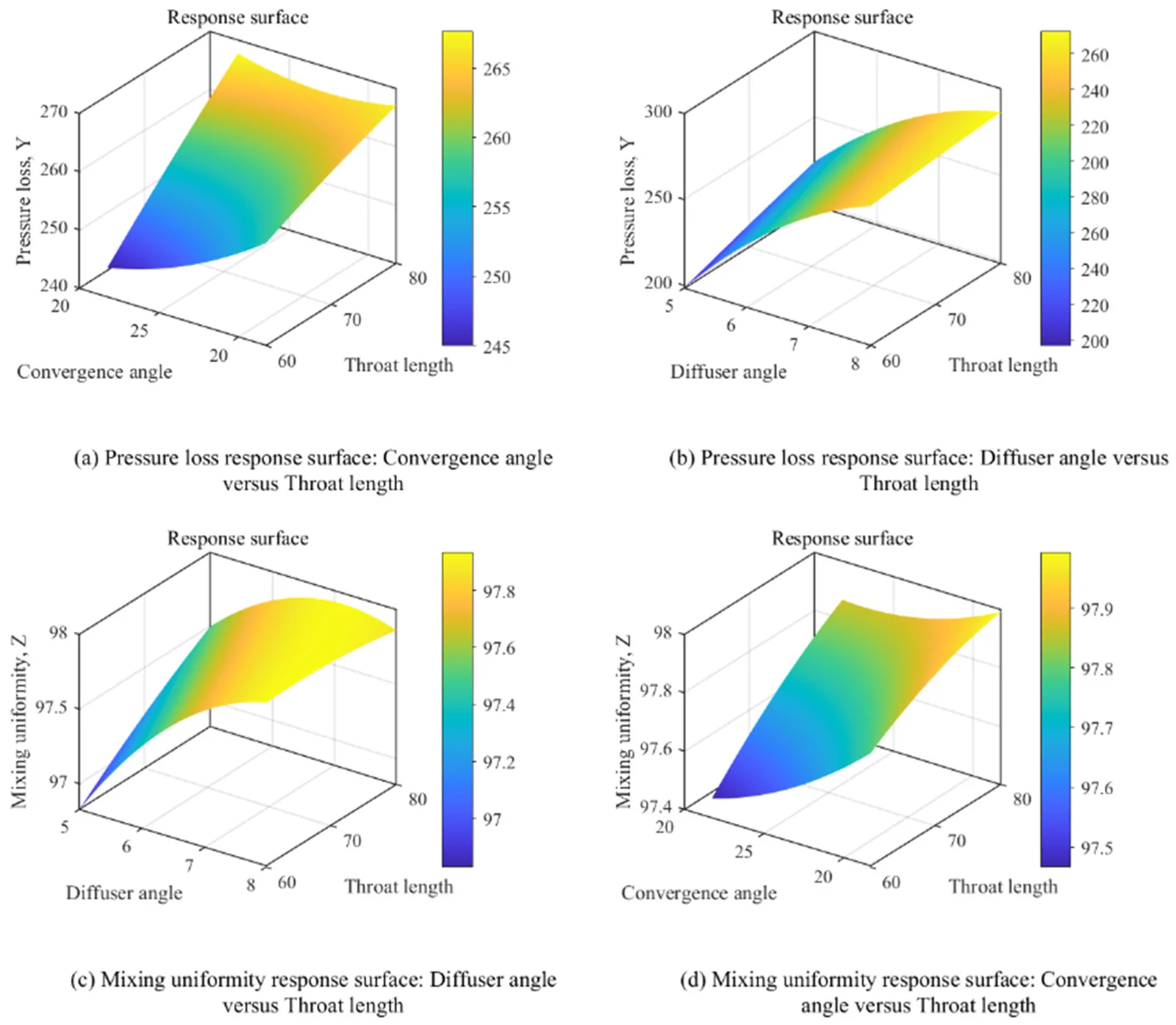
Typical three-dimensional response surfaces.

The three-dimensional response surfaces in Figure 18 show the interaction effects of geometric parameters on pressure loss. Contraction angle, throat length, and diffuser angle all affect pressure loss to different degrees, with diffuser angle being the most significant. Figure 18a indicates that at a fixed throat length, pressure loss increases slowly with contraction angle because local flow resistance in the contraction section increases. However, the overall variation is small, indicating that contraction angle has a limited influence on pressure loss. Figure 18b shows that pressure loss increases sharply with diffuser angle; this is because a larger diffuser angle tends to cause boundary-layer separation and local recirculation, intensifying flow-energy dissipation. Throat length also increases pressure loss to some extent, but its influence is weaker than that of diffuser angle.

The response surfaces for mixing uniformity show that diffuser angle and throat length are more influential than contraction angle. At a fixed throat length, increasing the contraction angle slightly decreases mixing uniformity, probably because overly concentrated flow weakens downstream disturbance development. As the diffuser angle increases, mixing uniformity improves substantially and reaches high levels at larger angles because the diffuser enhances shear, disturbance, and cross-sectional redistribution. Increasing throat length can also improve uniformity by extending the high-speed flow and species-exchange region. Overall, geometric parameters exhibit coupled effects: increasing diffuser angle enhances uniformity but increases pressure loss; increasing throat length promotes mixing but increases friction; and contraction angle has a relatively minor role. Thus, the structural optimization of the Venturi-type mixer is a trade-off design problem between high uniformity and low pressure loss.

#### 4.2.3. Optimization Results and Verification

**Figure 19:**
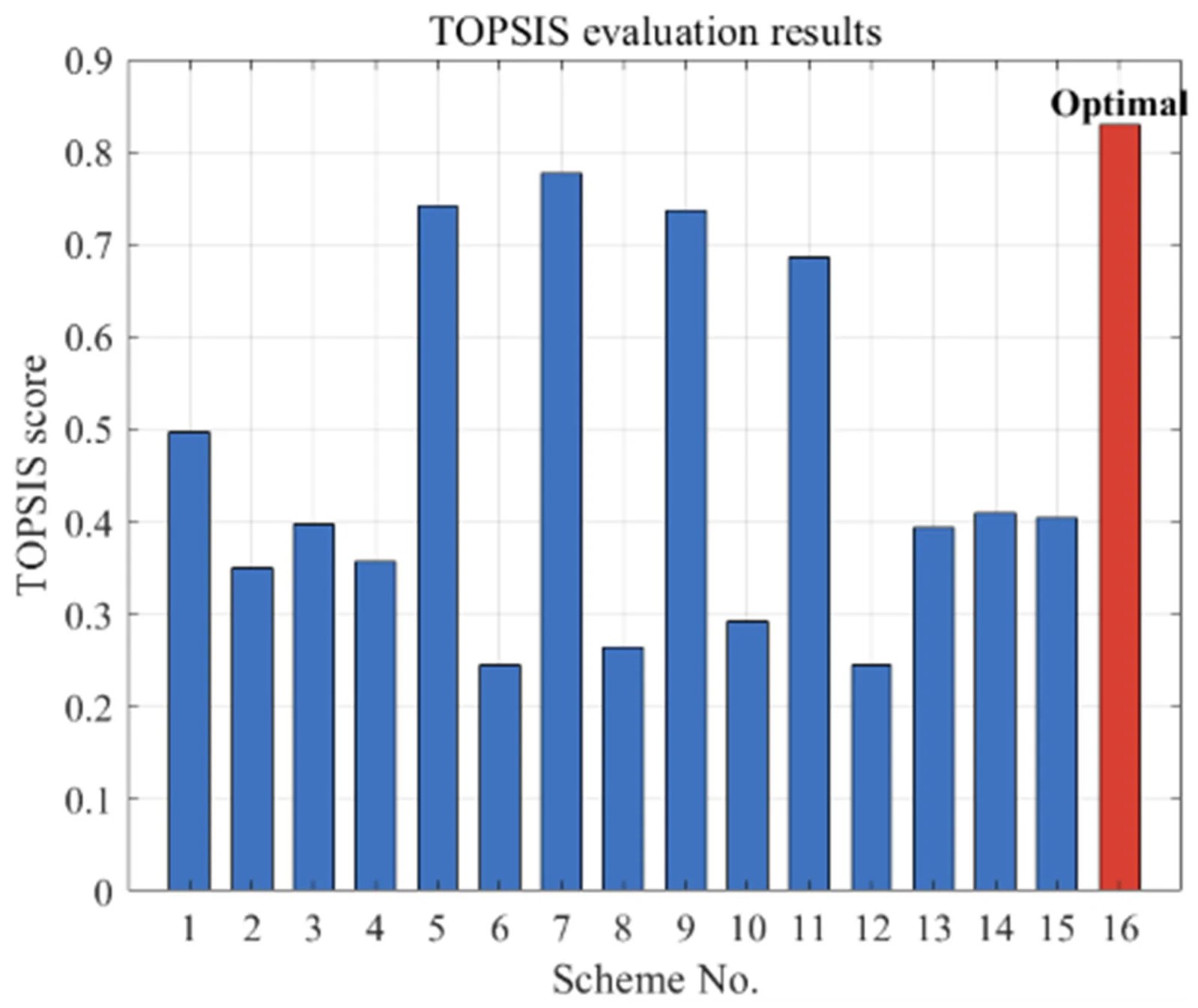
TOPSIS comprehensive performance evaluation index. The blue bars represent Schemes 1–15, while the red bar highlights the optimal Scheme 16.

To further verify the comprehensive superiority of the optimized scheme, TOPSIS was used to evaluate the candidate schemes. Pressure loss was regarded as a cost-type indicator, and mixing uniformity was regarded as a benefit-type indicator. With the weights determined above, a weighted normalized evaluation matrix was constructed. The Euclidean distances to the positive and negative ideal solutions were calculated, and the relative closeness was obtained. As shown on Table 12 and Figure 19, the results show that the 16th scheme, the CFD-validated optimal computational solution, has the highest comprehensive score, with a closeness coefficient of 0.8294 among all the 15 solution. The corresponding structural parameters are the optimized contraction angle, throat length, and diffuser angle.

### 4.3. Mixing-Mechanism Analysis

Overall, the mixing process in the Venturi-type mixer is not governed solely by molecular diffusion. Instead, it proceeds through continuous evolution, involving entrainment in the contraction section, high-speed shear in the throat, and large-scale entrainment and redistribution in the diffuser. The following section analyzes the mixing mechanism in the Venturi section under different hydrogen blending ratios using streamlines and hydrogen mole-fraction contours.

Based on the optimized computational solution, further analysis was conducted on the influence of fluid parameters on mixing uniformity. The hydrogen blending ratio characterizes both the hydrogen–natural gas proportion and the hydrogen inlet intensity, and therefore has a significant influence on internal flow organization, species transport, and final mixing uniformity. Previous studies indicate that hydrogen blending conditions modulate shear-layer development [1,16], hydrogen-jet diffusion range, and outlet homogenization. Therefore, a systematic analysis of hydrogen blending ratio was carried out based on the optimized structure.

With the natural-gas inlet velocity fixed at 10 m/s, the hydrogen inlet velocity was changed to adjust HBR and study hydrogen dispersion, mixing, and homogenization in the Venturi-type mixer. The calculated hydrogen inlet velocities corresponding to different HBR values are listed in Table 13.

#### 4.3.1. Effect of Hydrogen Blending Ratio on Mean Secondary-Flow Structures

**Figure 20:**
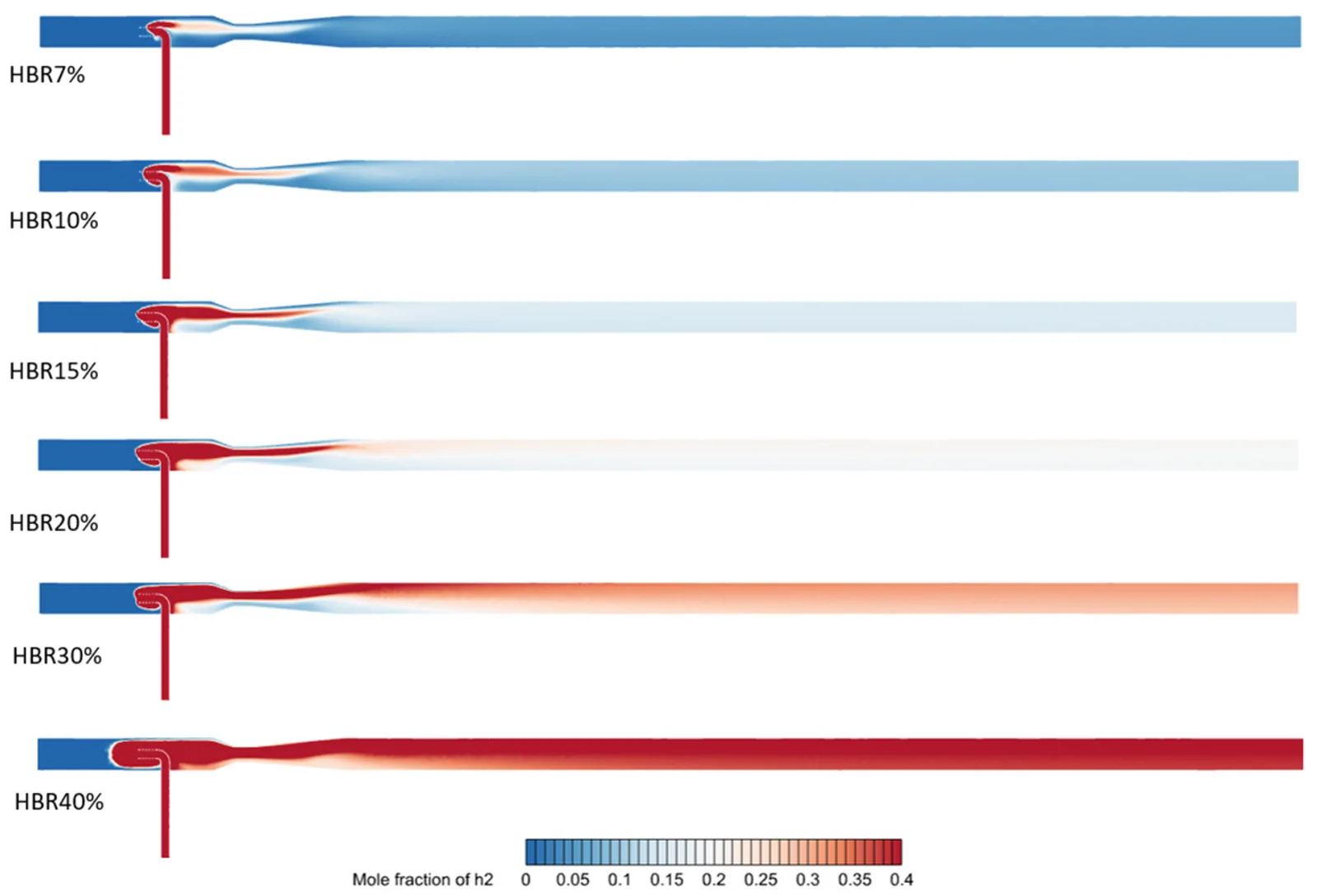
Hydrogen mole-fraction contours under different HBR conditions.

Figure 20 shows that as HBR increases, the flow pattern of hydrogen in the throat and downstream diffuser changes significantly. At low HBR, the hydrogen inlet velocity is low and the jet penetration capability is weak. Under the suction effect of the Venturi low-pressure region and the shear-mixing effect in the diffuser, hydrogen is rapidly entrained by the natural-gas main flow and diffuses circumferentially and radially. Thus, the downstream hydrogen-rich core is relatively short, and the composition distribution becomes more uniform. With increasing HBR, the hydrogen inlet velocity increases sharply, enhancing the axial momentum of the hydrogen jet and producing a more obvious high-concentration penetration zone. Local hydrogen-rich bands extend downstream, indicating stronger self-preservation of the high-speed jet and weakened transverse redistribution. Therefore, increasing hydrogen supply intensity does not necessarily improve uniformity; excessive inlet velocity may maintain stratification over a longer distance and delay homogenization.

**Figure 21:**
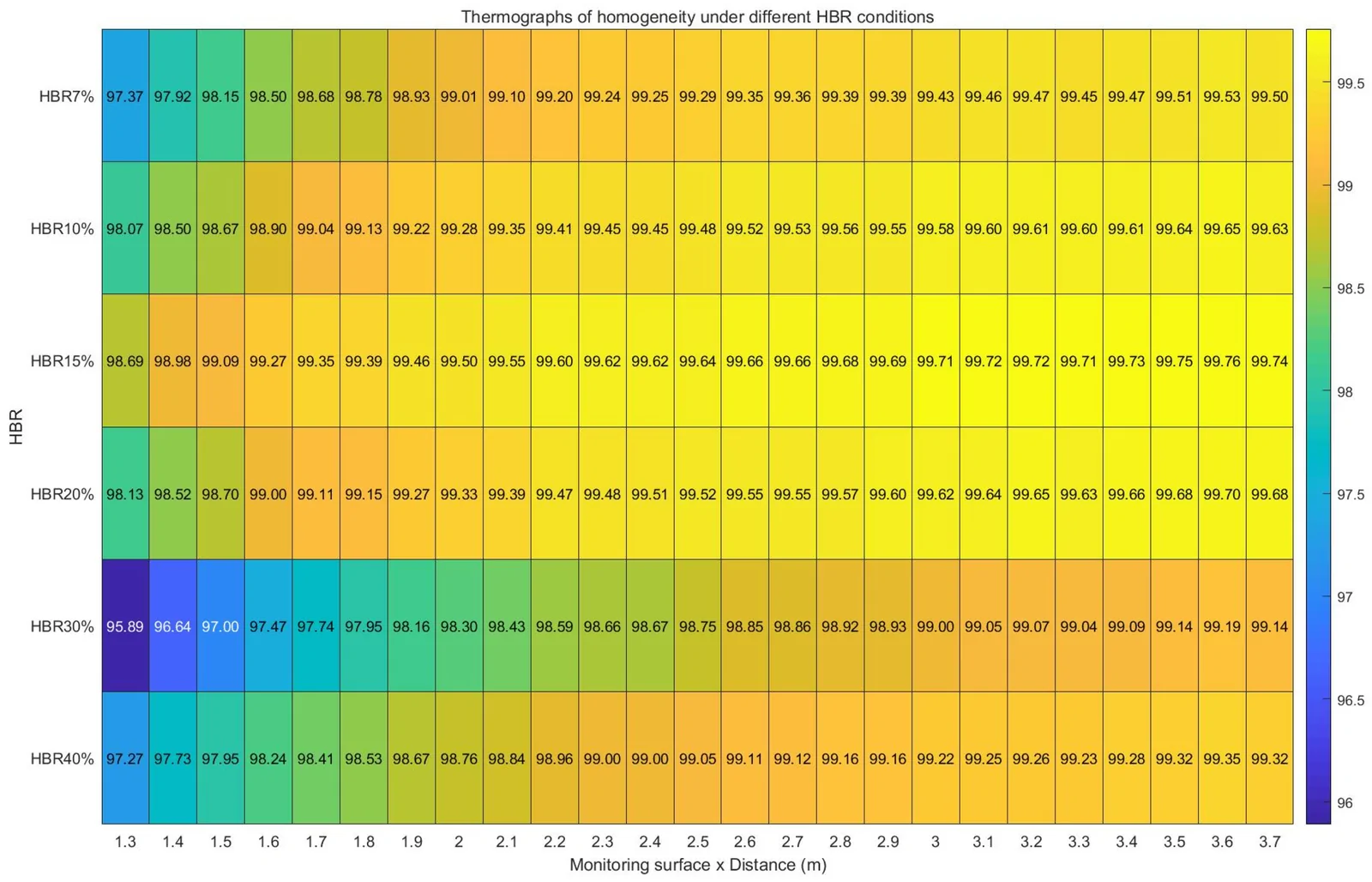
Heat map of mixing uniformity.

Figure 21 further shows that mixing uniformity generally increases with axial distance for all cases, demonstrating the continuous mixing-enhancement effect of the Venturi-type device. After hydrogen enters the main pipe, the high-speed throat region and diffuser expansion generate strong velocity and concentration gradients, and hydrogen diffuses progressively along the flow direction. HBR values of 7%, 10%, 15%, and 20% all exhibit high mixing uniformity, while HBR 15% shows the best comprehensive performance. At this condition, the uniformity reaches 98.69% at x = 1.3 m and increases to 99.74% at x = 3.7 m. This indicates a favorable momentum match between the hydrogen inlet and natural-gas main flow, providing sufficient entrainment and disturbance without causing serious jet bias or concentration stratification.

HBR 7% and HBR 10% also reach high downstream uniformity, but their early-stage mixing rates are slightly lower because hydrogen relies more on molecular and turbulent diffusion in the development section. When HBR increases to 20% or higher, the mixing behavior becomes more nonlinear. In particular, HBR 30% has the worst mixing performance, with a uniformity of only 95.89% at x = 1.3 m and a slower downstream growth rate. The contours indicate that a long high-concentration core forms downstream of the diffuser due to the strong inertia of the hydrogen jet, reducing cross-sectional redistribution efficiency.

Interestingly, when HBR is further increased to 40%, mixing uniformity recovers compared with HBR 30%, reaching 97.27% at x = 1.3 m and 99.32% at the final section. The extremely high hydrogen velocity promotes a more main-flow-like jet behavior, while the larger velocity difference also generates stronger shear and turbulence exchange, partly restoring transverse diffusion. Within the present range, HBR therefore exhibits a nonlinear trend of first increasing, then decreasing, and then partially recovering, with HBR 30% being an unfavorable interval and HBR 15% being a favorable interval.

Mechanistically, the Venturi mixer enhances hydrogen mixing through three processes: acceleration in the contraction section increases main-flow velocity and reduces static pressure, thereby strengthening hydrogen entrainment; strong velocity gradients near and downstream of the throat promote shear mixing between hydrogen and natural gas; and deceleration and redistribution in the diffuser provide space and time for homogenization. HBR essentially changes the momentum ratio between the hydrogen jet and natural-gas main flow, thereby altering flow organization, species-transport routes, and homogenization pathways.

#### 4.3.2. Effect of Hydrogen Blending Ratio on Mean Flow Structures

**Figure 22:**
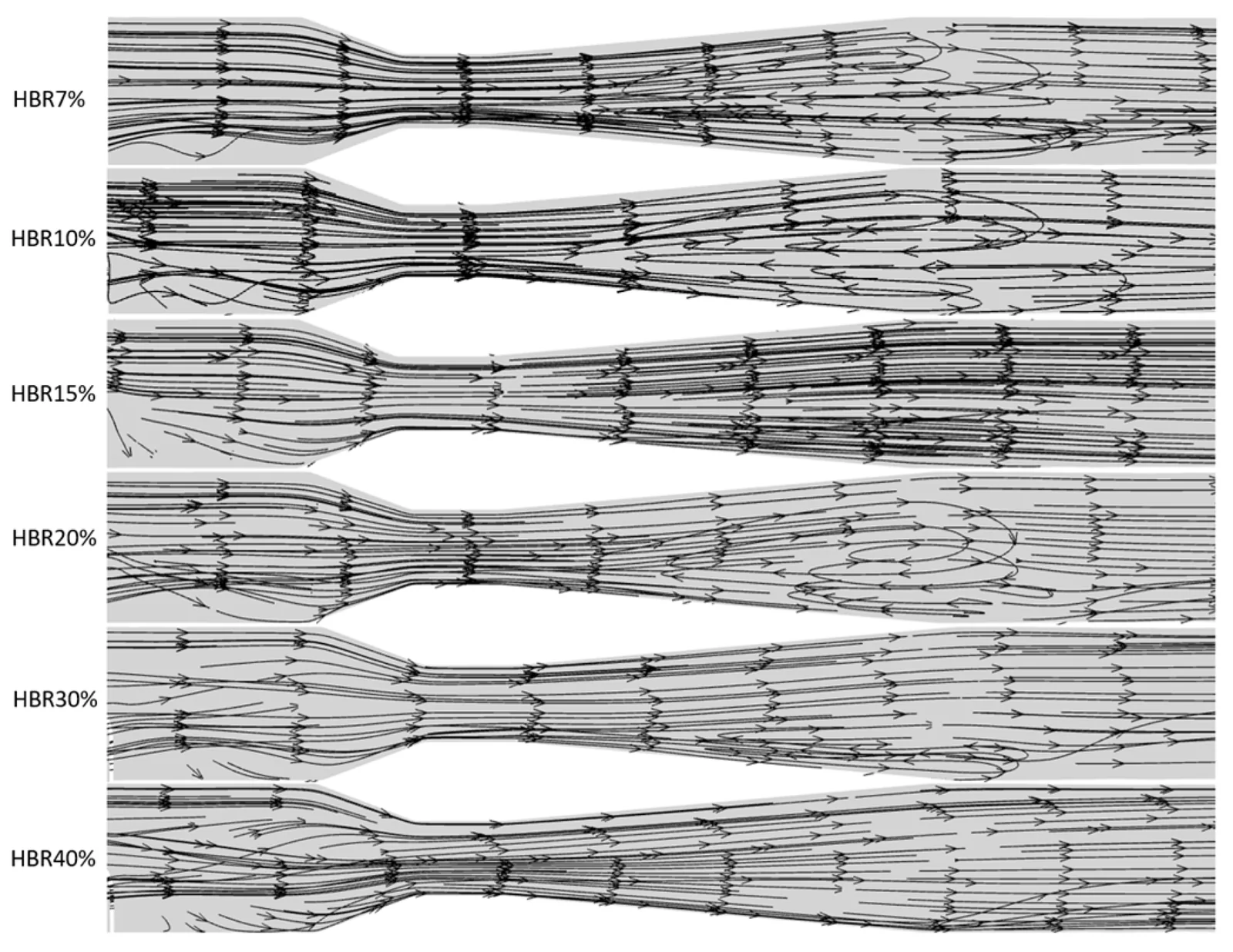
Three-dimensional mean streamline patterns at HBR values of range from 7% to 40%. The arrows indicate the local flow direction from the inlet (**left**) to the outlet (**right**). Apparent streamline crossings may occur because spatially separated three-dimensional streamlines around the wall are projected onto the two-dimensional viewing plane.

**Figure 23:**
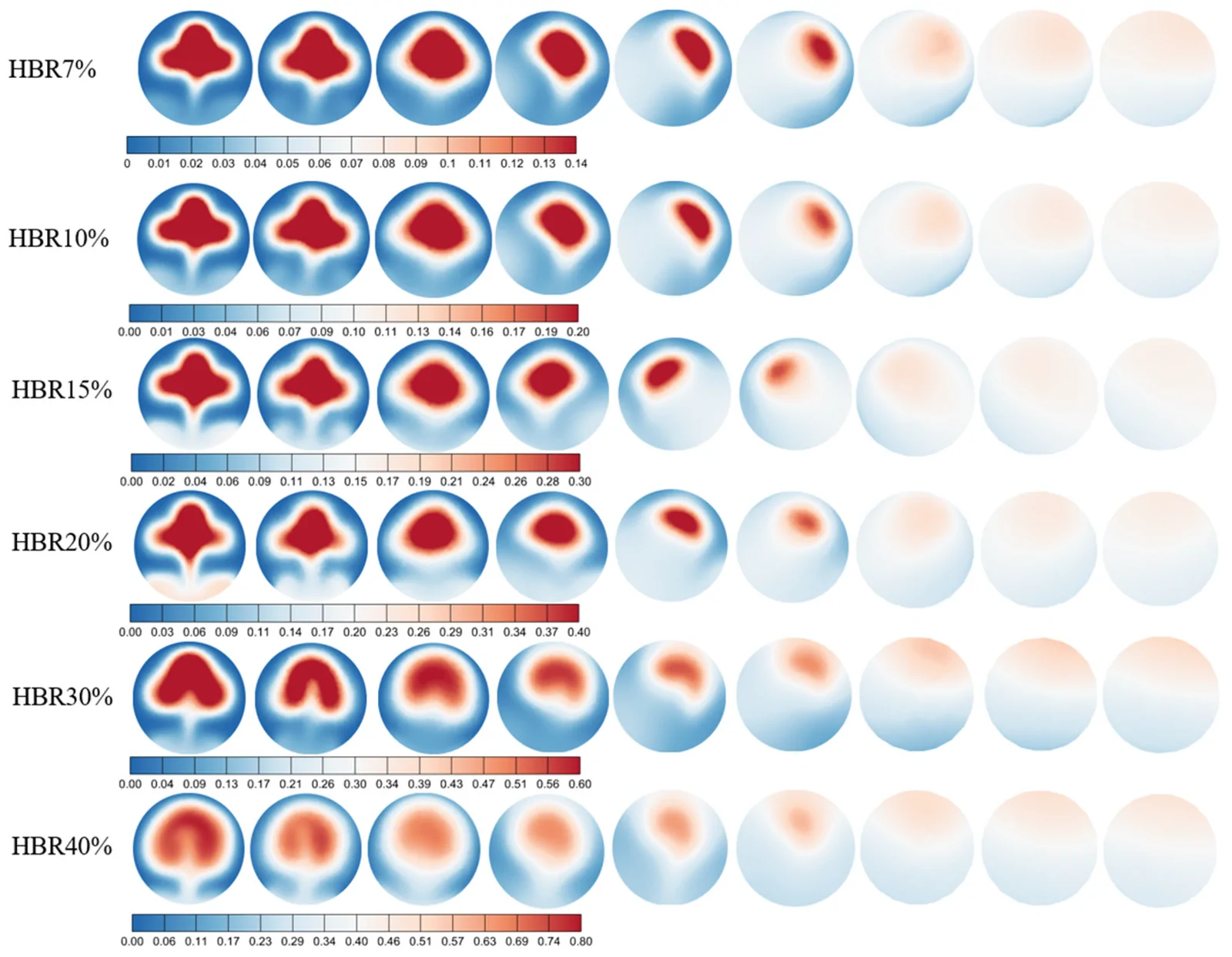
Sectional distributions in the Venturi mixing section under different HBR values.

Figure 22 and Figure 23 compare the statistically stationary mean-flow topology and species distributions obtained from independently converged steady-RANS simulations at different HBR values. Figure 22 and Figure 23 indicate that HBR affects not only species distribution but also the formation of secondary flow, recirculation zones, and local vortex cores downstream of the Venturi diffuser. As HBR increases, variation in mean vortex topology changes from weak local vortices to organized large-scale secondary flows and then to offset strong vortices or local mean closed-vortex regions.

Similar streamline-based analyses have been used in RANS or steady-flow studies of static mixers to interpret the relationship between mean recirculation structures, species redistribution, and pressure loss [17,34].

At low HBR, the hydrogen inlet velocity is low and the secondary jet weakly disturbs the main flow. The contraction section and throat are dominated by axial main flow, while only limited recirculation appears downstream of the diffuser. Vortices are mainly induced by the local shear layer where the hydrogen jet enters the main flow, and their size and range are limited to the injection neighborhood.

**Figure 24:**
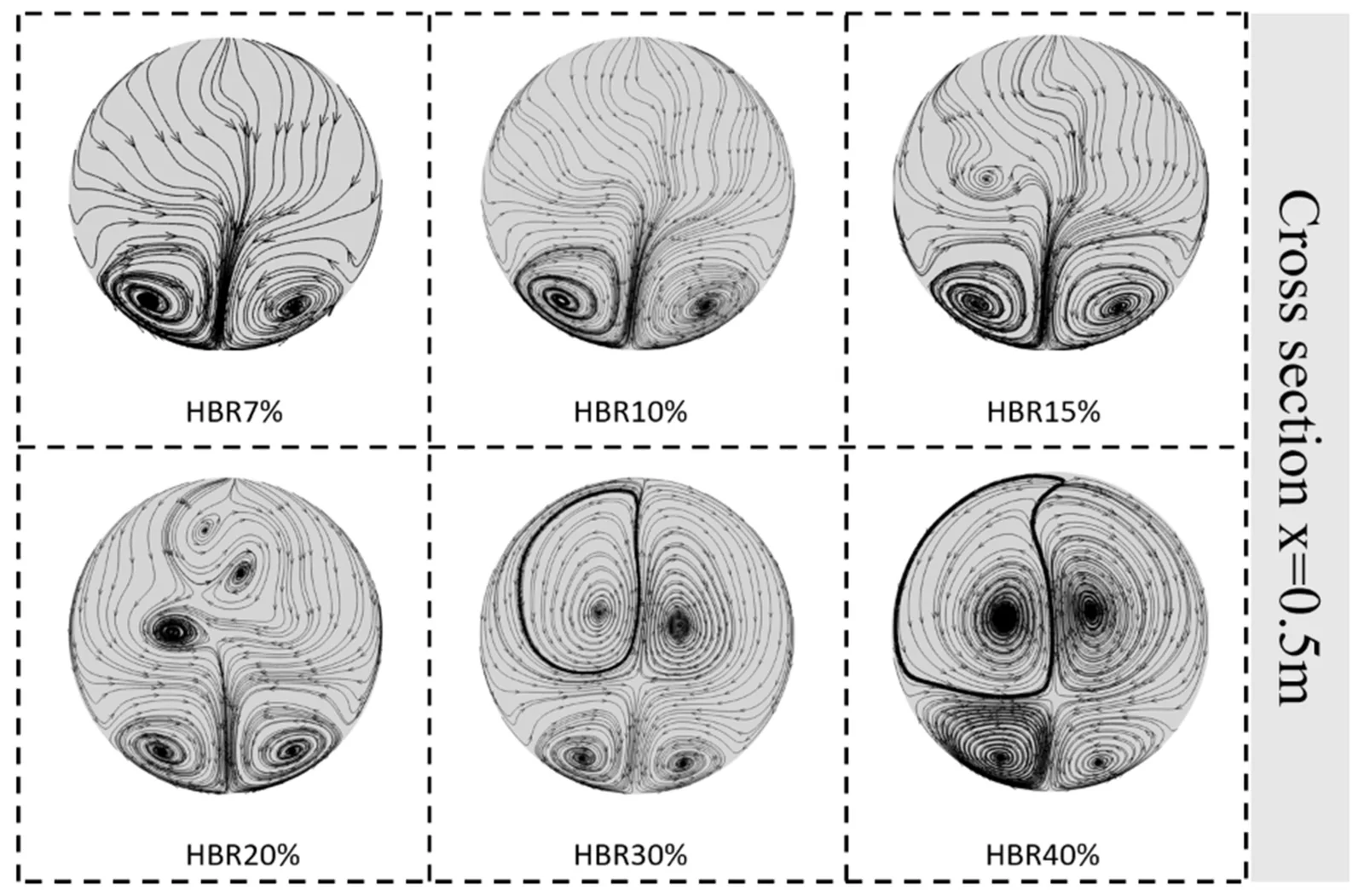
Time-averaged cross-sectional streamline patterns at x = 0.5 for HBR values of range from 7% to 40%. The arrows indicate the local direction of the in-plane secondary flow on the cross-section.

**Figure 25:**
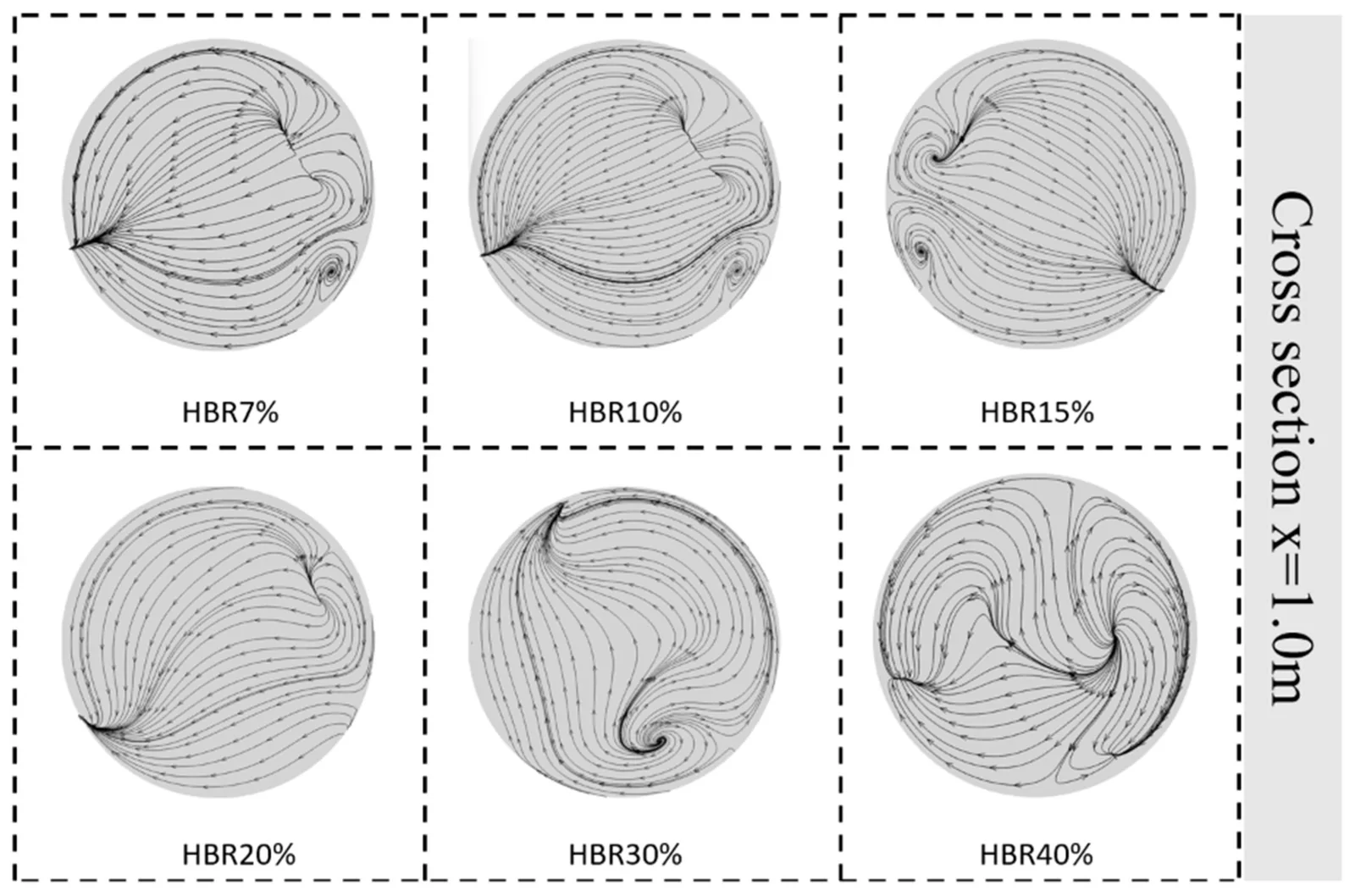
Time-averaged cross-sectional streamline patterns at x = 1.0 for HBR values of range from 7% to 40%. The arrows indicate the local direction of the in-plane secondary flow on the cross-section.

**Figure 26:**
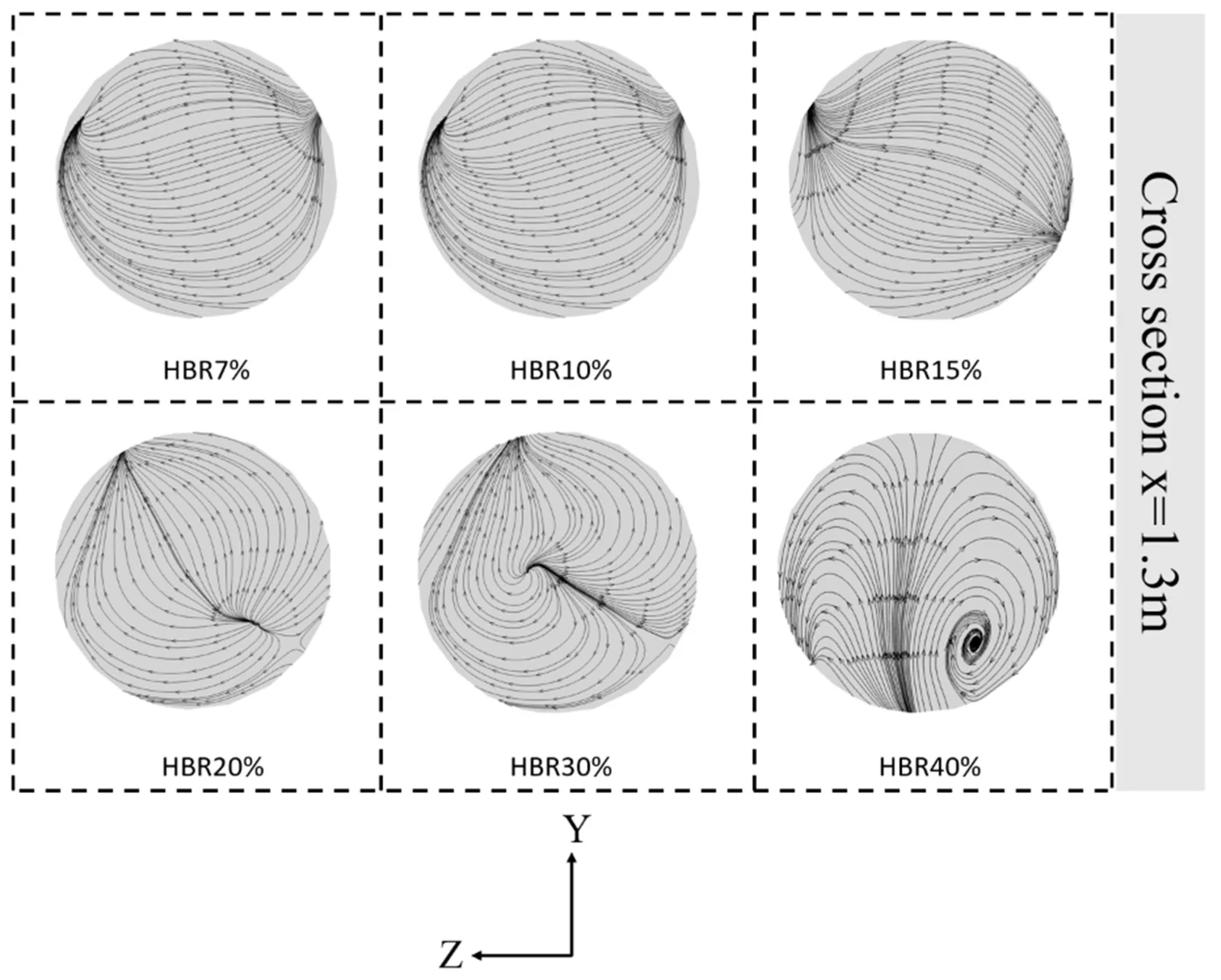
Time-averaged cross-sectional streamline patterns at x = 1.3 for HBR values of range from 7% to 40%. The arrows indicate the local direction of the in-plane secondary flow on the cross-section.

The cross-sectional mean streamline patterns in Figure 24, Figure 25 and Figure 26 show that when x = 0.5 m, HBR 7% and HBR 10% exhibit a clear bottom mean double-vortex structure. When x = 1.0 m and x = 1.3 m, the double vortices weaken and are replaced by a smoother overall circulation. This indicates that vortices at low HBR can promote local hydrogen entrainment but are neither strong nor persistent enough to drive full-section redistribution.

When HBR increases to the moderate range of 15–20%, mean vortex structures become more organized and persistent. The increased hydrogen inlet velocity strengthens the velocity difference between hydrogen and natural gas, enhancing shear-layer development downstream of the throat and diffuser. Longitudinal streamlines show stronger deflection, curling, and recirculation, implying the formation of stable large-scale secondary flows. In cross-sections, the bottom double vortices extend toward the center and interact with upper fluid, forming clearer circulation and stronger radial/circumferential transport.

The mole-fraction distributions further show that although hydrogen-rich regions still exist at moderate HBR, their shape changes from local accumulation to broader cross-sectional redistribution. In this stage, mean vortex structures no longer provide only local entrainment but also redistribute species over the whole section, which is most beneficial for mixing. Related studies also indicate that moderate hydrogen blending is favorable for persistent large-scale secondary and recirculating structures that enhance radial and circumferential transport [41].

At high HBR values of 30–40%, mean vortex structures become stronger, but their mixing function changes. The hydrogen-jet momentum increases rapidly, and jet penetration becomes dominant. The diffuser flow no longer consists only of organized secondary flows favorable to mixing but develops offset mean vortex cores and local mean closed-vortex zones. Such stronger vortices do not necessarily produce better mixing: hydrogen-rich fluid may be trapped in closed or semi-closed recirculation regions, and mean inter-vortex exchange may not increase correspondingly. Consequently, concentration stratification and sectional partitioning may become more evident.

In summary, the effect of HBR on mean vortex structures is staged and nonlinear. For the present Venturi-type mixer with the reverse hydrogen-injection tube, the flow entering the Venturi section is not an independently prescribed uniform inlet condition; it is affected by the wake, local separation, and asymmetric momentum redistribution induced by the hydrogen inlet tube. Under the present geometry and operating conditions, low HBR mainly produces local double vortices and weak recirculation with limited range; moderate HBR produces coordinated secondary flows and large-scale vortices that enhance radial and circumferential redistribution; and high HBR produces stronger but more biased or closed mean vortex structures that can maintain local stratification. HBR values in the moderate range, especially 15–20%, generate relatively organized secondary-flow structures in the examined cross-sections and are more favorable for radial and circumferential redistribution than the low- and high-HBR cases. Therefore, this conclusion should be interpreted as a case-specific observation for the present mixer configuration rather than a general rule for all Venturi-type hydrogen–natural gas mixers.

#### 4.3.3. Comparison Between the Optimized and Reference Central-Point Structure

**Figure 27:**
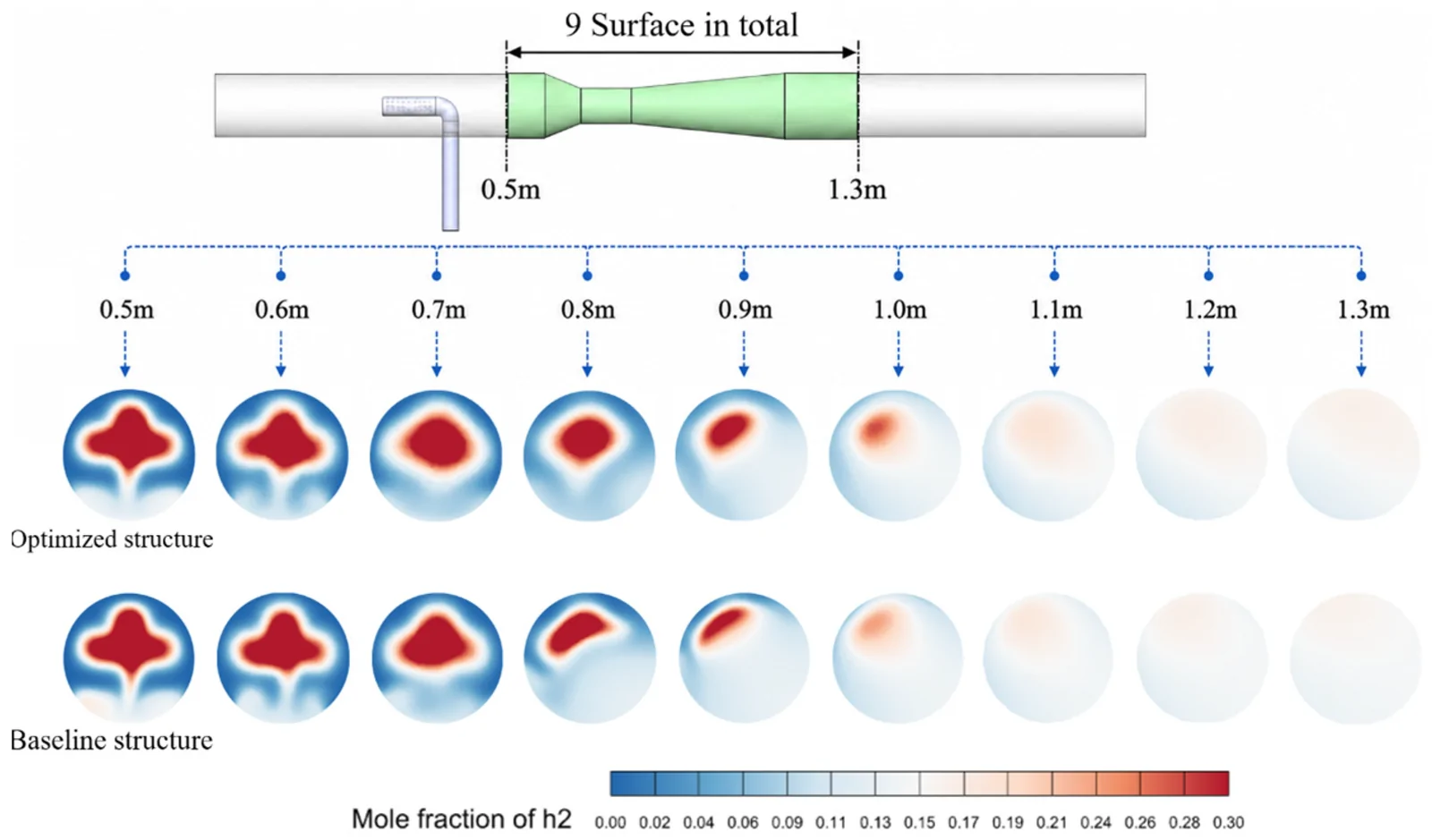
Comparison of optimization effects between the optimized and reference central-point structure under 15% HBR.

The numerical comparison between the two structures under 15% HBR condition is summarized in Table 14. Here, we chose 15% HBR to better illustrate the variation in hydrogen concentration in Figure 27, and also to demonstrate the capability of the optimized structure in different HBR situation.

Table 14 and Figure 27 show that the optimized structure does not simply pursue the highest mixing uniformity. Instead, it significantly reduces pressure loss while maintaining high mixing quality. The optimized parameters are a contraction angle of 20.7°, a throat length of 60 mm, and a diffuser angle of 5°. Compared with the original reference central-point structure, pressure loss decreases from 295.17 Pa to 237.66 Pa, a reduction of 57.51 Pa or approximately 19.48%. The final-section mixing uniformity decreases only from 99.81% to 99.54%, a reduction of 0.27 percentage points. Thus, the optimized structure achieves a large reduction in energy loss at the cost of only a very small decrease in mixing uniformity.

The section contours show that both the optimized and reference central-point structure exhibit a distinct hydrogen-rich core from 0.5 m to 0.8 m. As the flow develops downstream, the high-concentration region is sheared, stretched, and diffused by the main flow. After 1.0 m, the concentration gradients in both cases are greatly weakened, indicating sufficient mixing. In the optimized structure, the high-concentration region decays more smoothly near 0.9–1.0 m without obvious enlargement of local concentration clusters, indicating that the optimized geometry still maintains effective shear diffusion and transverse transport while reducing pressure loss.

From a mechanism perspective, reducing the contraction angle from 22.7° to 20.7° makes the geometric transition smoother. The velocity gradient remains present when the main flow enters the throat, but excessive local acceleration is avoided. Thus, additional loss and local disturbances in the contraction section decrease. Shortening the throat length from 70 to 60 mm reduces the wall-friction path in the throat while preserving a sufficient high-speed shear region for hydrogen entrainment and stretching. Reducing the diffuser angle from 6.5° to 5° makes pressure recovery smoother, weakens the adverse pressure gradient, and lowers the risk of flow separation, thereby reducing local energy loss and avoiding unstable concentration disturbances.

Overall, the optimized structure improves performance in three respects. First, pressure loss is substantially reduced, indicating effective control of flow resistance and local dissipation. Second, the final mixing uniformity remains above 99.5%, showing that the primary mixing performance is not sacrificed. Third, concentration evolution across sections is smoother, reflecting a continuous process of entrainment, shear, stretching, and diffusion through the contraction, throat, and diffuser. Therefore, the advantage of the optimized structure lies in achieving a better compromise between high mixing uniformity and low pressure loss. Under 7% HBR, it maintains sufficient mixing while reducing pressure loss by nearly 20%, demonstrating superior comprehensive performance compared with the reference central-point structure design.

## 5. Conclusions and Future Outlook

To evaluate the application value of a Venturi-type mixing device in hydrogen-enriched natural-gas pipelines, this study selected three structural parameters and one fluid parameter as research variables. Single-factor simulations were conducted for each structural parameter, and a three-level BBD matrix with 15 cases was designed to analyze parameter interactions. Entropy weighting, a genetic algorithm, and TOPSIS were used for multi-indicator optimization and verification. Based on the optimized structure, the influence of hydrogen blending ratio was further investigated, and the optimized and reference central-point structure were compared. The main conclusions are as follows:

(1) Numerical simulations show that the Venturi-type mixer improves mixing by amplifying asymmetric momentum exchange and inducing secondary-flow and streamwise-mean flow structures. Reasonable combinations of structural and fluid parameters can control vortex intensity and thereby reduce unnecessary energy loss during hydrogen-enriched natural-gas transport.
(2) Single-factor analysis indicates that among the three structural parameters, the diffuser angle has the strongest influence on both mixing uniformity and energy loss. A too-small diffuser angle provides insufficient radial redistribution and poor mixing, whereas a too-large diffuser angle strengthens the adverse pressure gradient, local recirculation, and boundary-layer separation. Therefore, structural parameters clearly reflect the trade-off between mixing enhancement and pressure loss, and their coupled effects must be considered in optimization.
(3) The entropy-weighted GA-TOPSIS optimization identified the optimal structural parameters of the Venturi-type mixer within the prescribed design range as a contraction angle of 20.7°, a throat length of 60 mm, and a diffuser angle of 5°. The CFD verification of this optimized structure gives a pressure loss of 206 Pa and a final-section mixing uniformity of 97.43%, indicating that the optimized geometry maintains high mixing quality while controlling flow energy loss.
(4) This study demonstrates the feasibility of using a Venturi-type mixing device to enhance hydrogen–natural gas mixing. However, the present flow-field analysis mainly relies on qualitative visualization from steady-state numerical simulations, and variation in mean vortex topology has not yet been quantified. Future work should include experimental validation and more refined numerical simulations, which will provide more reliable guidance for practical engineering applications.

The present steady-RANS framework is suitable for evaluating the time-averaged mixing uniformity and pressure-loss characteristics required for geometric screening and response-surface optimization. However, it cannot directly resolve instantaneous shear-layer development, coherent vortex motion, scalar intermittency, or concentration-fluctuation spectra.

Large-eddy simulation has been shown to provide detailed unsteady information for turbulent jet mixing by resolving large-scale turbulent motions while modeling subgrid-scale transport [42]. In our future work, LES or a hybrid RANS–LES method will therefore be applied to selected representative configurations, including the reference central-point structure, the optimized structure, under operating conditions corresponding to both favorable and unfavorable hydrogen blending ratios. The comparisons will include time-averaged mixing uniformity and pressure loss, hydrogen-concentration fluctuations, scalar variance, resolved turbulent kinetic energy, vortex-identification criteria, and pressure and concentration spectra. The sensitivity to physically realistic time-dependent inlet turbulence will also be examined, followed by experimental validation of the predicted mean and fluctuating quantities.

## Figures and Tables

**Table 1:** Main geometric parameters of the mixing device.

Geometric Parameter	Symbol	Value (mm/°)
Main natural-gas pipe diameter	$D_{n}$	100
Distance from hydrogen pipe to inlet	$L_{1}$	400
Distance from hydrogen pipe to contraction section	$L_{2}$	100
Venturi mixing-section length	$L_{v}$	500
Hydrogen injection-pipe penetration depth	$L_{in}$	60
Outlet final-mixing section length	$L_{3}$	3000
Hydrogen injection-pipe diameter	$D_{h}$	25
Hydrogen injection elbow radius	$R_{h}$	25
Hydrogen injection section length	$L_{hi}$	60
Venturi contraction angle	A	18.7°, 20.7°, 22.7°, 24.7°, 26.7°
Venturi throat length	B	50, 60, 70, 80, 90
Venturi diffuser angle	C	4°, 5°, 6.5°, 8°, 9°

**Table 2:** Summary of the numerical models, operating conditions, and boundary-condition settings.

Category	Item	Setting
Software	CFD software	ANSYS Fluent 2024 R1
Solver setup	Solver type	Pressure-based
Solver setup	Velocity formulation	Absolute
Solver setup	Simulation type	Steady-state
Solver setup	Pressure–velocity coupling	Coupled
Solver setup	Initialization method	Hybrid
Physical model	Turbulence model	Standard k-epsilon model
Operating condition	Operating pressure	101,325 Pa
Thermal condition	Inlet temperature	300 K
Natural-gas inlet	Boundary type	Velocity inlet
Natural-gas inlet	Inlet velocity	10 m/s
Hydrogen inlet	Boundary type	Velocity inlet
Hydrogen inlet	Inlet velocities	12.04 m/s
Outlet boundary	Boundary type	Pressure outlet
Outlet boundary	Outlet gauge pressure	0 Pa
Wall boundary	Momentum condition	Stationary no-slip wall
Body force	Gravity	9.81 m/s^2^ in the negative y-direction
Near-wall treatment	Wall treatment	Standard wall functions
Convergence control	Residual convergence criterion	10^−6^

**Table 3:** Spatial discretization schemes adopted in the numerical simulations.

Term	Scheme
Gradient reconstruction	Least-squares cell-based
Pressure discretization	Second-order
Momentum equations	Second-order upwind
Energy equation	Second-order upwind
Species transport equations	Second-order upwind
Turbulent kinetic energy, k	First-order upwind
Turbulent dissipation rate, ε	First-order upwind

**Table 4:** Grid-independence verification parameters of the mixing device.

Mesh Level	GCI Grid	Cells	Refinement Ratio	Minimum Orthogonal Quality
Fine	Grid 1	617,032	—	0.1
Adopted	Grid 2	233,028	$r_{21}=1.38$	0.1
Coarse	Grid 3	95,069	$r_{32}=1.34$	0.1

**Table 5:** Calculations result of discretization error.

Quantity	Definition	$\phi_{3}$	$\phi_{2}$	$\phi_{1}$	p	$\phi_{ext}$	$GCI_{21}$	$GCI_{32}$	Asymptotic Ratio
COV	Plane 13	0.0209	0.0227	0.021	1.59	0.019	9.37%	16.21%	1.03
Pressure loss	Reported Net	239.022	259.81	259.27	12.15	259.26	0.0052%	0.27%	1.01

**Table 6:** Single-factor test scheme for contraction angle.

Case No.	Contraction Angle/Deg	Throat Length/mm	Diffuser Angle/Deg
A1	18.7	70	6.5
A2	20.7	70	6.5
A3	22.7	70	6.5
A4	24.7	70	6.5
A5	26.7	70	6.5

**Table 7:** Single-factor test scheme for throat length.

Case No.	Contraction Angle/Deg	Throat Length/mm	Diffuser Angle/Deg
B1	22.7	50	6.5
B2	22.7	60	6.5
B3	22.7	70	6.5
B4	22.7	80	6.5
B5	22.7	90	6.5

**Table 8:** Single-factor test scheme for diffuser angle.

Case No.	Contraction Angle/Deg	Throat Length/mm	Diffuser Angle/Deg
C1	22.7	70	4.0
C2	22.7	70	5.0
C3	22.7	70	6.5
C4	22.7	70	8.0
C5	22.7	70	9.0

**Table 9:** Parameter ranges for response-surface optimization.

Case No.	Influencing Factors	Symbols	Level		
			−1	0	+1
1	Contraction angle	A	20.7	22.7	24.7
2	Throat length	B	60	70	80
3	Diffuser angle	C	5	6.5	8

**Table 10:** Final Box–Behnken experimental matrix and response values. (Scheme 16 is the GA-derived optimization-verification case. It was not included in the original 15-run Box–Behnken design or in response-surface model fitting.).

Scheme No.	Contraction Angle	Throat Length	Diffuser Angle	Pressure Loss	Mixing Uniformity μ (%)
1	20.7	60	6.5	245.06	97.48
2	20.7	80	6.5	267.34	97.93
3	24.7	60	6.5	257.95	97.71
4	24.7	80	6.5	267.01	97.97
5	20.7	70	5	208.98	97.04
6	20.7	70	8	283.76	97.83
7	24.7	70	5	215.59	97.48
8	24.7	70	8	288.84	98.03
9	22.7	60	5	199.30	96.82
10	22.7	60	8	278.51	97.98
11	22.7	80	5	225.20	97.44
12	22.7	80	8	285.54	97.86
13	22.7	70	6.5	258.25	97.71
14	22.7	70	6.5	256.68	97.72
15	22.7	70	6.5	258.94	97.85
16	20.7	60	5	206.36	97.43

**Table 11:** ANOVA for COV and Δp.

	Sum of Squares		Mean Square		F-Value		*p*-Value	
Source	COV(%)	Δp (Pa)	COV(%)	Δp (Pa)	COV(%)	Δp (Pa)	COV(%)	Δp (Pa)
Model	1.6833	11,487.8	0.187	1276.42	22.54	526.61	0.0016	<0.0001
x_1_	0.1021	73.5896	0.1021	73.58	12.3	30.36	0.0172	0.0027
x_2_	0.1813	516.415	0.1813	516.41	21.85	213.05	0.0055	<0.0001
x_3_	1.0676	10,337.6	1.0676	10,337.6	128.66	4265	<0.0001	<0.0001
x_1_x_2_	0.0096	43.6974	0.0096	43.69	1.15	18.02	0.3311	0.0081
x_1_x_3_	0.0152	0.5836	0.0152	0.58	1.83	0.24	0.2338	0.64
x_2_x_3_	0.136	89.0462	0.136	89.04	16.39	36.73	0.0098	0.0018
x_1_^2^	0.0066	11.5972	0.0066	11.59	0.79	4.78	0.4135	0.08
x_2_^2^	0.0031	0.5528	0.0031	0.55	0.37	0.22	0.5669	0.65
x_3_^2^	0.1579	402.002	0.1579	402	19.02	165.85	0.0073	<0.0001
Residual	0.0415	12.1191	0.0083	2.423	-	-	-	-
Lack of fit	0.0301	9.4201	0.01	3.14	1.77	2.32	0.3813	0.31
Pure error	0.0114	2.6991	0.0057	1.3495	-	-	-	-

**Table 12:** CFD verification of the optimized structure comparison with the reference central-point structure.

Performance Parameter	Original Central-Point Structure	Optimized Structure, CFD-Validated
Contraction angle/deg	22.70	20.7
Throat length/mm	70.00	60
Diffuser angle/deg	6.50	5
Pressure loss/Pa	258.94	206.36
Final-section mixing uniformity/%	97.85	97.43
TOPSIS closeness coefficient	0.41	0.82

**Table 13:** Hydrogen inlet velocities corresponding to different HBR values.

Hydrogen Blending Ratio, HBR	Hydrogen Inlet Velocity (m/s)
7%	12.04
10%	17.78
15%	28.24
20%	40.00
30%	68.57
40%	106.67

**Table 14:** Optimization results under 15% HBR.

Performance Parameter	Original Baseline Structure	Optimized Structure, CFD-Validated
Contraction angle/deg	22.70	20.7
Throat length/mm	70.00	60
Diffuser angle/deg	6.50	5
Pressure loss/Pa	295.17	237.66
Final-section mixing uniformity/%	99.81	99.54

## Data Availability

The key data and source code supporting the findings of this study are openly available in https://github.com/character76/Performance-analysis-and-optimization-of-a-Venturi-Type-Hydrogen-Natural-gas-mixer-supporting-data (accessed on 16 July 2026).
